# Sex‐Specific Methylomic and Transcriptomic Responses of the Avian Pineal Gland to Unpredictable Illumination Patterns

**DOI:** 10.1111/jpi.70040

**Published:** 2025-03-17

**Authors:** Fábio Pértille, Tejaswi Badam, Nina Mitheiss, Pia Løtvedt, Emmanouil Tsakoumis, Mika Gustafsson, Luiz Lehmann Coutinho, Per Jensen, Carlos Guerrero‐Bosagna

**Affiliations:** ^1^ Department of Organismal Biology Physiology and Environmental Toxicology Uppsala University Uppsala Sweden; ^2^ Department of Computational Biology Luxembourg Centre for Systems Biomedicine University of Luxembourg Esch‐sur‐Alzette Luxembourg; ^3^ IFM Bioinformatics Linköping University Linköping Sweden; ^4^ Avian Behavioural Genomics and Physiology Group IFM Biology Linköping University Linköping Sweden; ^5^ Animal Biotechnology Laboratory, Animal Science and Pastures Department “Luiz de Queiroz” College of Agriculture (ESALQ) University of São Paulo (USP) Piracicaba São Paulo Brazil

**Keywords:** circadian, epigenetics, gene expression, illumination, light, methylome, miRNA, pineal gland, sex differences, transcriptomic

## Abstract

In the production environment of chickens, exposure to unpredictable light patterns is a common painless stressor, widely used to influence growth rate and egg production efficiency. The pineal gland, a key regulator of circadian rhythms through melatonin secretion, responds to environmental light cues, and its function is modulated by epigenetic mechanisms. In this study, we investigated how the pineal gland methylome and transcriptome (including micro‐RNAs) interact to respond to a rearing exposure to unpredictable illumination patterns, with a particular focus on sex differences. We conducted an integrative multi‐omic analysis—including methylomic (MeDIP‐seq), transcriptomic (RNA‐seq), and miRNA expression profiling—on the pineal gland of Hy‐Line White chickens (*n* = 34, 18 females, 16 males) exposed to either a standard 12:12 light–dark cycle (control) or a randomized, unpredictable light schedule from Days 3 to 24 post‐hatch. Our findings reveal that unpredictable light exposure alters the pineal gland methylome and transcriptome in a sex‐specific manner. However, while transcriptomic differences between sexes increased due to the stress, methylomic differences decreased, particularly on the Z chromosome. These changes were driven by females (the heterogametic sex in birds), which became more male‐like in their pineal methylome after exposure to the illumination stress, leading to reduced epigenetic sexual dimorphism while maintaining differences at the gene expression level. Further, we implemented a fixed sex effect model as a biological proof of concept, identifying a network of 12 key core genes interacting with 102 other genes, all linked to circadian regulation and stress adaptation. This network of genes comprises a core regulatory framework for circadian response. Additionally, tissue‐specific expression analysis and cell‐type specific expression analysis revealed enrichment in brain regions critical for circadian function, including neuronal populations involved in circadian regulation and the hypothalamic–pituitary–thyroid axis. Together, these findings provide strong evidence of sex‐specific epigenetic transcriptomic responses of the pineal gland upon illumination stress and offer valuable insights into the interplay of different omic levels in relation to circadian response.

## 1 Introduction and Background

In the chicken production environment, exposure to unpredictable illumination patterns is a prominent, non‐invasive stressor [1], widely employed to maximize mass accretion [2, 3] and egg production [4]. Beyond its economic implications, light exposure in vertebrates affects a broad range of physiological outcomes, including behavior, metabolism [5, 6], and reproduction [7]. A crucial region of vertebrate brains involved in the regulation of circadian rhythmicity is the pineal gland, which depending on the species, directly or indirectly responds to patterns of light exposure [8, 9], owing to the structural and functional differences in circadian regulation among vertebrates. In mammals, the pineal gland is regulated via the sympathetic nervous system by two hypothalamic regions, the suprachiasmatic nucleus (SCN), which is the main controller of circadian rhythmicity and connected to photic stimuli via the retina in the hypothalamus, and the paraventricular nucleus, which responds to the SCN [10, 11]. In contrast, the bird pineal gland responds directly to photic signals from the retina [12] and the calvaria, subsequently stimulating pinealocytes [13, 14], functioning as an independent circadian oscillator in response to light exposures [15] that stimulates autoregulatory transcriptional loops [16].

In vertebrates, the main hormone secreted by the pineal gland is melatonin [9]. Circulating levels of melatonin peak at night and dip during the day [17], serving as a critical endogenous oscillator in the circadian system [18]. Circadian melatonin synthesis in chickens starts during the embryonic phase [19], and its oscillation affects a variety of physiological processes such as reproductive performance [20], weight gain, and sexual maturity [21]. Conversely, artificial illumination can disturb this circadian clock, leading to melatonin suppression, rhythm disruption, and phase shifts [22]. Beyond these circadian alterations, unpredictable illumination effectively induces physiological and behavioral stress in poultry without causing physical harm [23, 24, 25]. Because the molecular mechanisms underlying the circadian clock involve transcriptional gene regulation via exposure to light [22, 26], there might be an involvement of epigenetic mechanisms in its regulation. Epigenetic processes such as methylation, hydroxymethylation of CpG dinucleotides, histone modifications, miRNA interactions, and chromatin condensation states [27, 28] are sensitive to external stimuli and able to regulate gene expression in the long term [14, 29]. Such epigenetic control is crucial during early development and significantly influences adult phenotypes [30]. DNA methylation influences gene expression by modulating interactions with chromatin proteins and transcription factors [29, 31]. Additionally, the transcription process is intricately influenced by both pre‐ and posttranscriptional elements [32]. miRNAs are pivotal in RNA degradation and translational repression, engaging in RNA interference pathways that influence chromatin state and gene expression [33, 34, 35, 36, 37].

Recent studies have demonstrated that environmental stressors can induce long‐term transcriptomic modifications in pinealocytes, the primary cells of the pineal gland. For instance, neonatal hypoxic‐ischemic brain damage (HIBD) has been shown to cause subtype conversions between β‐pinealocytes and α‐pinealocytes, accompanied by distinct transcriptomic changes [38]. Additionally, single‐cell RNA sequencing of the mammalian pineal gland has identified two distinct pinealocyte subtypes exhibiting cell type‐specific daily patterns of gene expression, indicating that epigenetic mechanisms may play a role in regulating pinealocyte function and, consequently, melatonin production [39]. Transcriptomic profiling of the pineal gland in sheep has further revealed differentially expressed long non‐coding RNAs and genes related to reproduction, providing insights into how gene expression regulation in the pineal gland might influence reproductive traits [40]. Similarly, dynamic transcriptome analysis in pigs highlighted the expression profiles of mRNAs and long non‐coding RNAs during postnatal development, emphasizing the regulatory roles of lncRNAs in pineal gland development and function [41]. Also, histone modifications and DNA methylation have been shown to be important players in the regulation of circadian rhythms [29] by influencing the ability of the pineal to respond to environmental light cues [27, 42]. These findings suggest that environmental factors can lead to transcriptional and epigenetic changes in pinealocytes, potentially impacting circadian rhythm regulation.

Evidence across vertebrates shows that epigenetic and transcriptional responses to environmental stimuli are sex dependent, particularly in the brain [43]. For example, in mammals, sex differences are well reported in normal microglia, from embryo to adulthood [44], and are shaped by sex chromosomes and sex hormones [45]. Sex‐specific transcriptomes have been identified in sexually dimorphic brain nuclei, suggesting that environmental factors interact with genetic programming to influence brain function in a sex‐dependent manner [31]. Particularly, research in birds has advanced our understanding of how transcriptional and epigenetic mechanisms modulate sexual differentiation in brain and behavior [33]. In songbirds, for example, male and female brains exhibit distinct transcriptional profiles that align with behavioral and cognitive differences [34]. Shorebirds, in turn, display sex differences in immune gene expression in the brain, linking transcriptional variation to physiological and behavioral traits [35]. In chickens, we have previously shown that a 5‐generation selection of animals for high or low fear of humans resulted in large sex‐specific differences in DNA methylation in the hypothalamus [36].

Despite the known lifelong phenotypic impacts of light‐induced stress, the epigenetic responses of the pineal gland's nuclear genome to fluctuating light patterns remain largely unexplored [37]. Here, we exposed chickens to unpredictable illumination patterns during rearing and then performed an integrative analysis of DNA methylation, miRNA, and gene expression to elucidate the complex molecular interplay involved in the pineal response to light stimuli in birds. Importantly, we investigated these molecular responses in both sexes, to identify potential sex‐specific patterns. While recent research from our group has investigated the effects of light exposure stress on mitochondrial DNA methylation and mito‐nuclear interactions [46], our present study focuses on nuclear mechanisms. Our study sheds light on the nuanced interplay between light exposure and sex‐dependent epigenetic and gene expression modifications in regulating the pineal response of birds, with important ramifications for understanding circadian regulation across species.

## 2 Materials and Methods

The handling and maintenance of chickens used in this study were conducted under the permission of the Regional Committee for Scientific Research on Animals, (License Nr. 50‐13 Linköping).

### 2.1 Animals

Fertilized Hy‐Line white leghorn chicken (*Gallus gallus domesticus*) eggs, sourced from Swedfarm, Linghem, Sweden, were incubated at 37.8°C in a 60% humidified atmosphere, with hourly rotation until hatching. Post‐hatching, chicks were wing‐tagged, placed in boxes, and then transferred to cages housing mixed‐sex groups of five to six chickens each. Because the White Leghorn hybrid used in this study does not exhibit sex‐specific feathering at hatch, groups were not sex‐balanced before culling. Throughout the study, all birds had unlimited access to water and food, with wood chips lining each cage floor for comfort. Initially, feed trays containing starter food were provided, later replaced by hanging feeders dispensing Pennafood. To ensure optimal growth conditions, the chicks were also provided with heated roofing. All were reared in cages where both temperature and lighting, provided by light‐emitting diode (LED) sources, were controlled (Supporting Information S7: Figure S1).

### 2.2 Experimental Design

**Figure 1:**
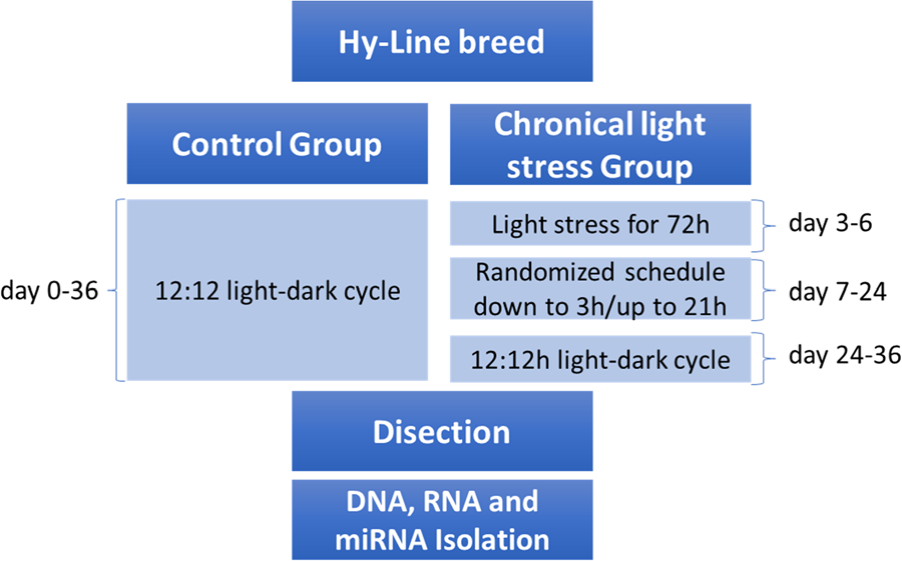
Treatment process of randomly divided newly hatched chickens. The control group was kept at a standard 12:12 light–dark cycle for their entire life. The chronic light stress group was exposed to unpredictable illumination patterns on days 7–24 of age.

After 1 day of incubation, the chicks were randomly allocated into two groups. The control group was maintained under a consistent 12:12 light–dark cycle throughout the experiment. Conversely, the chronic light stress group underwent exposure to unpredictable illumination patterns in two phases—a method previously shown to induce stress responses in poultry without causing physical harm [23, 24, 25]. First, from days 3 to 6 chicks experienced continuous light for 72 h, disrupting early circadian entrainment. Then, from days 7 to 24 they were exposed to a randomized illumination schedule in which the duration of light exposure varied daily between 3 and 21 h. To control for overall light exposure, the total amount of light and dark periods was balanced with that of the control group over every 3‐day cycle. Starting from day 25 and for the duration of the study, the randomized illumination patterns were reverted to the standard 12:12 light‐dark cycle, aligning with the control group's condition (Figure 1).

The illumination stress was administered using 2 W LED Spotlights (Ljuskoncept D5014) mounted on the roof of each cage (0.57 m height). Each spotlight emitted a warm white light at 3000 K, with an intensity of 200 lumens and a 70‐degree beam angle. The illumination schedule was precisely managed via timers to deliver unpredictable exposure patterns while ensuring equal cumulative light exposure over 3‐day cycles compared to the control group. To prevent light spill and maintain the integrity of experimental conditions, cages were effectively isolated, with preliminary light measurements confirming no cross‐cage illumination. At the age of 34–36 days, a scheduled culling process was conducted over 3 days, whereupon chickens from both the control and chronic illumination stress groups were humanely euthanized by decapitation. Subsequently, pineal glands were carefully extracted, immediately snap‐frozen in liquid nitrogen to preserve RNA and DNA integrity for downstream analysis. Sex was determined post‐mortem by gonadal inspection. All collected data were retained for comprehensive analysis, with no individual subject's results omitted. Importantly, the primary aim of this study was to investigate epigenetic and transcriptomic changes resulting from exposure to illumination stress, rather than assessing circadian rhythmicity. Due to the number of individuals in the study and the multiple samples collected, the collection time took a few days. Because the sample collection was balanced timewise between experimental groups, we do not expect impacts on the main aim, which was the sustained effects of stress, rather than transient, time‐of‐day‐dependent changes.

### 2.3 DNA and RNA Isolation

To isolate total RNA, miRNA, and DNA from pineal gland tissue, Allprep DNA/RNA/miRNA Universal kit (Qiagen GmbH, Germany) was used. First, the complete pineal gland was homogenized in 350 μL RLT Plus buffer (Allprep DNA/RNA/miRNA Universal kit, Qiagen GmbH, Germany), which contained guanidine thiocyanate for the tissue lysis and then homogenized for 40 s in a rotor‐stator homogenizer (FastPrep‐24 Homogenizer, MPTM Biomedical, California, USA). Further steps were performed according to the manufacturer's protocol. The RNA concentration was determined by Nanodrop spectrophotometer and Gene Quant pro RNA/DNA calculator Software (NanoDrop ND‐1000 Spectrophotometer, Thermo Scientific, Wilmington, USA). RNA and miRNA samples were stored at −80°C or directly synthesized to complementary DNA (cDNA), whichwas stored at −20°C, together with the DNA samples.

### 2.4 Methylation and RNA Sequencing Methodology

*DNA Methylation Sequencing (MeDIP‐Seq):* DNA was extracted from pineal gland samples and subjected to methylated DNA immunoprecipitation sequencing (MeDIP‐Seq). We used 8 μg of DNA, sheared to an average size of 350 bp. The sheared DNA then underwent immunoprecipitation using a 5‐methylcytosine antibody (Diagenode). whole‐genome PCR amplified with the WGA2 kit from Sigma‐Aldrich, cleaned with the QIA quick PCR purification kit from Promega, and sent to sequencing to identify differentially methylated regions (DMRs).

*RNA and mRNA Sequencing:* Total RNA, including mRNA, was isolated from the same pineal gland samples. For mRNA, we synthesized cDNA using a Reverse Transcription System kit employing Oligo(dT) primers (Maxima H Minus Double Stranded cDNA synthesis kit, Thermo Scientific) For miRNA and other non‐coding RNAs, reverse transcription was performed using the TaqMan Micro RNA reverse transcription Kit (Applied Biosystems). Subsequent library preparation followed the protocol optimized for the Ion Torrent sequencing platform. The libraries were sequenced to obtain short single‐end reads, capturing the transcriptional landscape and allowing for the identification of differentially expressed genes (DEGs) and miRNAs.

The sequencing processes for both DNA methylation and RNA profiles were conducted using the Ion ProtonTM System for high‐throughput, next‐generation sequencing. All sample preparations and sequencing were performed at the SNP&SEQ Technology Platform in the Science for Life Laboratory (SciLifeLab), Sweden, ensuring high‐quality data for subsequent analyses.

### 2.5 Bioinformatics Analysis

#### 2.5.1 Raw Data Analysis

Sequencing reads were quality‐filtered with Torrent Suite Software (v4.0) and aligned using TMAP, with FastQC v.0.11.3 checking read quality. Analysis across omics data utilized a linear model with five contrasts to differentiate phenotypic chicken groups, detailed in Table 1.

**Table 1:** Overview of linear model contrasts for phenotypic group comparison.

Contrast	Group1	Group2	Purpose
MSvFS	Male stress	Female stress	Sex differences on stress group
MCvFC	Male control	Female control	Sex differences on control group
MSvMC	Male stress	Male control	Stress effect on male
FSvFC	Female stress	Female control	Stress effect on female
SvC	Stress (male and female)	Control (male and female)	Stress effect regardless to the sex

*Note:* “Stress” represents the individuals exposed to unpredictable light schedule, which were considered as the chronic light stress group.

#### 2.5.2 Gene Expression Profile Analysis

Sequencing reads were initially preprocessed using Trimmomatic v. 0.32 [47] to trim bases with a quality score below 20, retaining only those with a Phred score above 33 and a minimum length of 36 bps. The reads were then aligned to the chicken reference genome GRCg6a, sourced from Ensembl, using RNA‐Star v. 2.4.1d [48] for precise mapping. Transcript assembly and quantification of the RNA‐Seq reads against this reference genome were conducted using StringTie‐1.3.5 [49]. To compile a comprehensive matrix of read counts mapped to specific genomic features, we employed the prepDE.py Python script provided by “StringTie” (https://github.com/gpertea/stringtie/blob/master/prepDE.py).

#### 2.5.3 Screening of Differentially Expressed MicroRNAs (DMiR)

Reads were preprocessed by filtering out reads shorter than 17 bases using Trimmomatic v. 0.32 [47], and retaining only reads with quality score of all positions ≥ 20 bps. Reads were mapped to reference built from mature miR sequences extracted from miRBase version 21 using BBMap v. 34.33 (http://sourceforge.net/projects/bbmap/). Additionally, reads were mapped to the reference genome and summarized at a gene biotype level (using parameter ‐g gene_biotype) using featureCounts from subread package v. 1.4.6 [50]. A matrix summarizing read counts for specific genomic features was created, counting all uniquely matched reads to each miRNA, inclusive of mismatches, indels, and non‐template 3′‐end extensions.

#### 2.5.4 Screening of DMRs

Quality‐trimmed reads were aligned to the chicken reference genome (Gallus_gallus 6.0, NCBI) using the Bowtie2 tool (v.2‐2.3.4.2) [51] with its default settings. The resulting alignment files were converted to BAM format for downstream analysis. Indexing and assessing the coverage depth of these BAM files were accomplished using Samtools (v.0.1.19) [52], employing the “index” and “depth” functions.

Unlike the default setting of 300 bp windows in the MEDIPS package, our analysis focused on regions of interest (ROIs) defined by a merged BED file [53]. This file was generated using the MACS peak calling program [54], incorporating the results from the four predefined contrasts (SMvF, CMvF, MSvC, FSvC) under study. The resulting MEDIPS analysis output—a matrix of read counts for each ROI—served as the basis for further investigation.

#### 2.5.5 Omic Data Integration

Analysis was conducted in R version 3.6.1 [55], setting a read count threshold of at least four per region across the three screenings to ensure representation in each of the four contrasts (MSvFS, MCvFC, MSvMC, FSvFC). Read counts were normalized using the voom function from the limma package for mean‐variance modeling at the observational level. Differential analysis for the five specified contrasts (MSvFS, MCvFC, MSvMC, FSvFC, and SvC) was performed using the linear models approach within the limma package, as outlined in Table 1.

In our study, we explored three omic layers:

1. Transcriptomics: DEGs from mRNA,
2. Regulomics: target‐genes from DMiR,
3. Methylomics: DMRs.

To integrate these data, we aligned each omic layer to the protein‐coding gene level. For DMiRs, we matched miRDB_v6 for *G. gallus* targets using the get_multimir function from the Bioconductor multiMiR package, expanding the DMiR list to include all corresponding target genes. Methylomic analysis involved extracting DMR coordinates and associated gene annotations using the org.Gg.eg.db package, extending genomic range coordinates by 5 and 10 kb to encapsulate regulatory and potential enhancer regions. These DMRs were further annotated using the annotatePeak function from the ChIPseeker package [56], utilizing gg_txdb for transcript metadata and org.Gg.eg.db for the chicken genome annotations.

To describe the molecular functions, cellular components, and biological processes of each gene, we employed the enrichGO function for gene ontology (GO) and the Kyoto Encyclopedia of Genes and Genomes (KEGG) for molecular interaction networks, applying the compareCluster function in ChIPseeker for analysis.

To enhance visualization of the biological functions impacted by our data‐associated genes, we created word clouds depicting functional pathway terms alongside the genes involved in each pathway, utilizing the tool available at https://www.jasondavies.com/wordcloud/.

#### 2.5.6 Module Analysis

To dissect and pinpoint co‐regulated genes or those with physical interactions, we employed the DIAMoND function within the MODifieR package in R [57], utilizing the high‐confidence protein–protein interaction (PPI) network [58] from STRING v.11 [59] (score > 700) as our foundation. This network comprised 8366 unique genes and 214 694 interactions, facilitating the identification of interconnected gene modules. The resulting highly interactive gene list was visualized as networks through Cytoscape v3.6.2 [60]. We further conducted functional enrichment analysis on these modules using g:Profiler [61], applying an FDR‐adjusted *p*‐value cutoff of < 0.05 to ensure significance.

## 3 Results

### 3.1 General Genomic Sequence Parameters

In our multi‐omic analysis, we evaluated 14 492 genes for differential expression across all contrasts used in this study (Table 1). These genes were located within 10 kb from the 37 382 windows scanned to detect DMRs. Additionally, we analyzed 100 085 predicted gene targets from the 352 microRNAs (miRDB.org) scanned to detect DMiR. This integrative approach enabled a comprehensive analysis that used genes as a unified reference across the transcriptomic, methylomic, and regulomic levels. For the raw data analysis, before normalization, a minimum read count threshold of four was applied to ensure that each sample group included three individuals, thereby guaranteeing at least one count per contrast for the tree omic features analyzed (Supporting Information S10: Table S1).

### 3.2 Multidimensional Scaling (MDS) Exploring Sex and Treatment‐Specific Clustering Across Multi‐Omic Analyses

**Figure 2:**
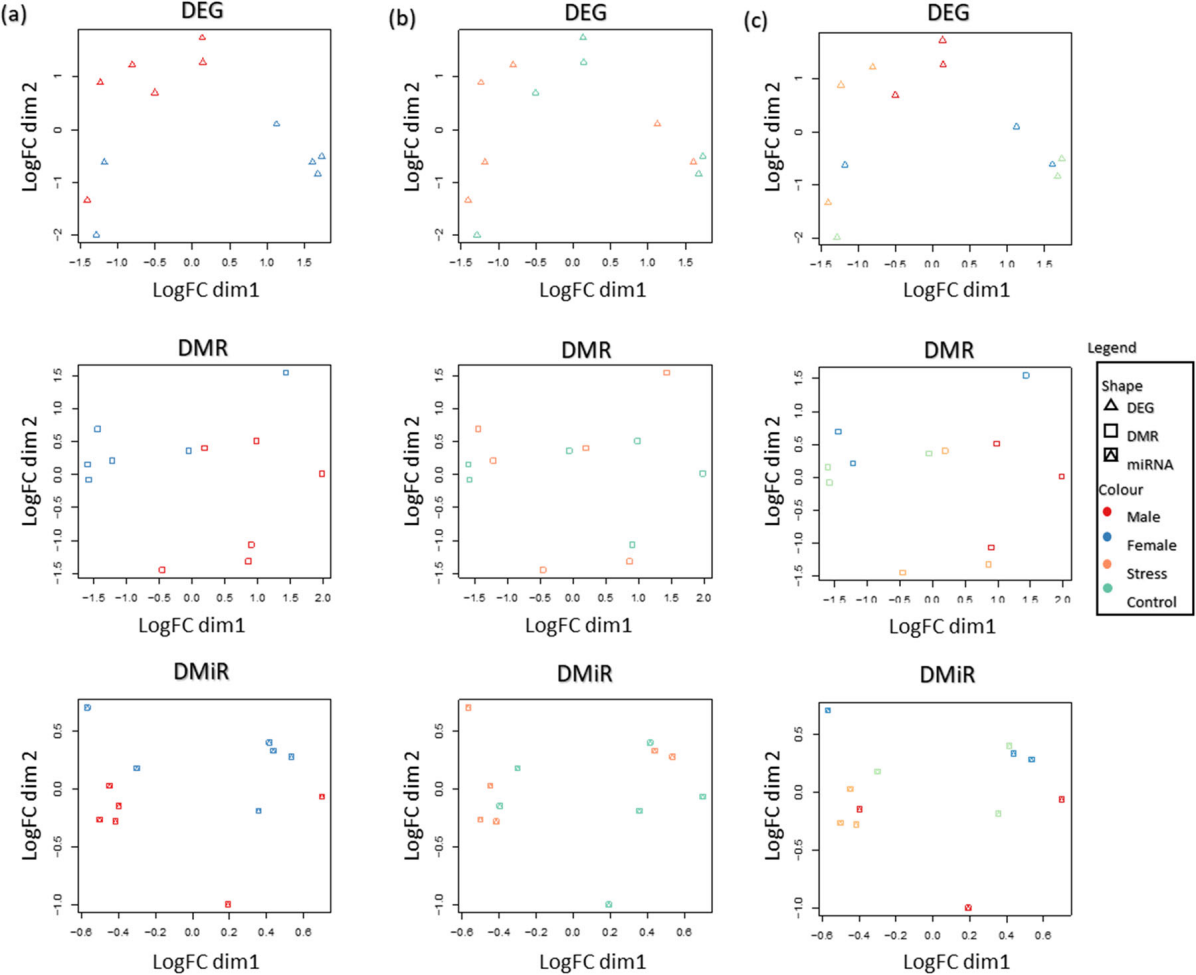
Representation of multidimensional scaling (MDS) analysis highlighting distinctions based on sex (a; left), treatment (b; center), and their combination (c; right), through the integration of DEG (top), DMR (center), and DMiR (bottom) data. As object in the analysis, we used the genes from DEG (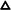), windows from DMR (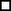), and miRNA from DMiR (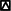) analysis.

We performed MDS on the total features obtained per omic analysis (Supporting Information S10: Table S1) to uncover potential cluster formation between sexes, treated versus control groups, and their combinations (Figure 2). Sex differences were pronounced at all three omic levels. In the combined analysis, distinct clustering was observed between the female and stress groups, and between the male and control groups for both the DEG and DMR analyses.

### 3.3 Omic Integration and Statistical Contrasts

**Figure 3:**
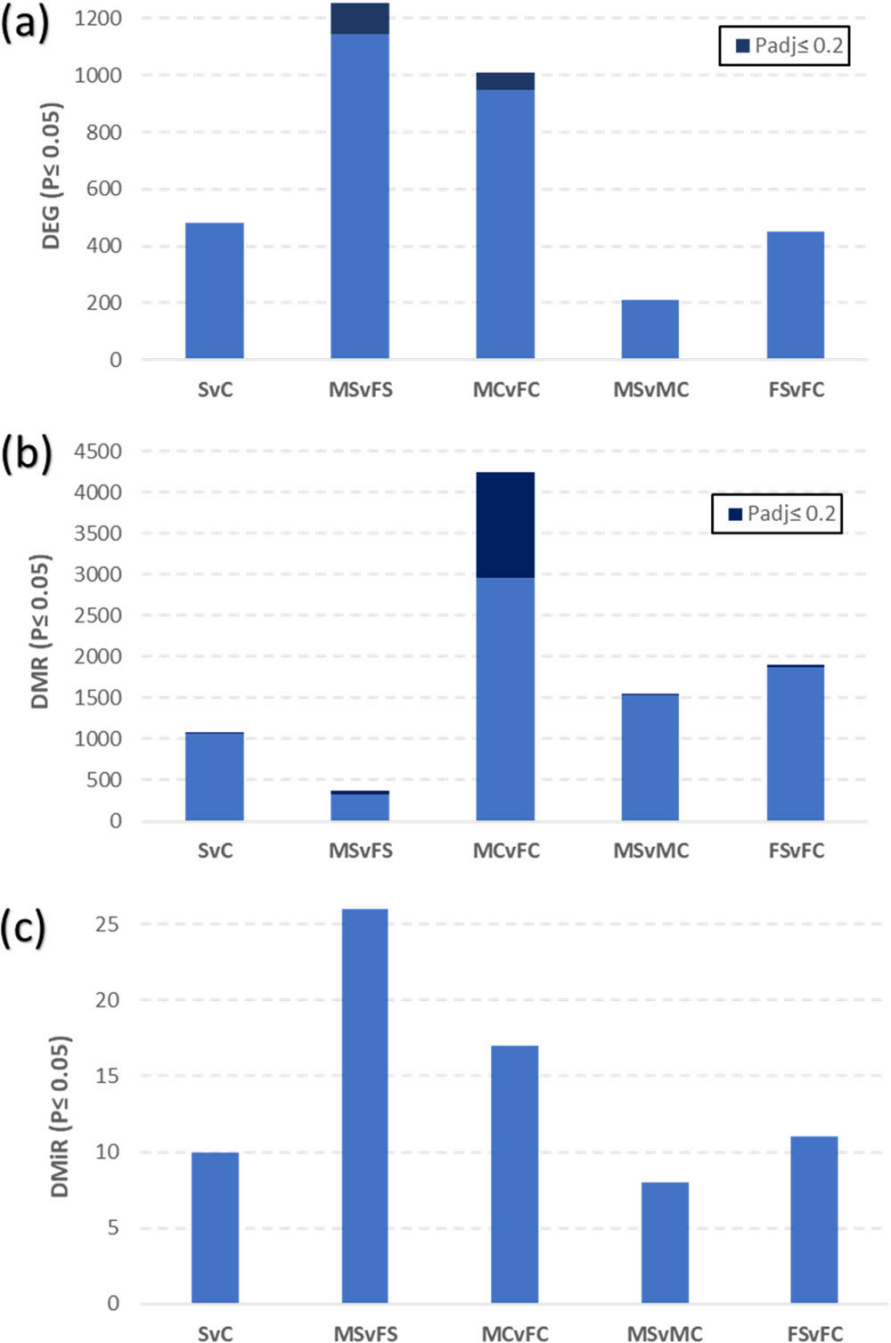
Number of differentially expressed genes (DEG) (a), differentially methylated regions (DMRs) (b), and differentially expressed microRNAs (DMiR) (c) across the five contrasts analyzed in the study (SvC, MSvFS, MCvFC, MSvMC, FSvFC), with a significance threshold of *p* ≤ 0.05.

Our data were analyzed using five linear model contrasts (Table 1). We established two significance thresholds for the analysis: a standard threshold (*p* ≤ 0.05) for a broader view, and a more stringent threshold (FDR‐adjusted *p* ≤ 0.2) for gene‐specific effects (Supporting Information S10: Table S1). For the analysis of miRNA‐predicted target genes, while two confidence thresholds are presented, only those with a prediction score greater than 90% were considered for further analyses. To describe the findings (presented in Supporting Information S10: Table S1), we divided our results into two separate sections: Section 3.4, which focuses on the four initial contrasts (MSvFS, MCvFC, MSvMC, and FSvFC), and Section 3.5, which examines the effect of the treatment while using sex as a fixed effect in the model (SvC). The number of markers identified in each contrast is shown in Figure 3.

### 3.4 Sex Differences

We observed important sex effects in all contrasts between sexes, both in controls and stress groups. However, while transcriptomic differences (including MiRs) were increased between sexes in the Illumination stress contrast (MSvFS) compared to controls (MCvFC), methylomic differences between sexes drastically decreased when comparing the same groups (Figure 3).

**Figure 4:**
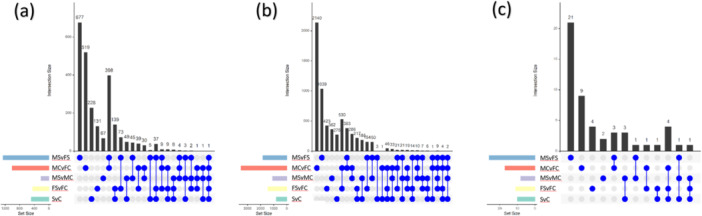
Barplot illustrating the distribution of omic features across the 5 contrasts (MSvFS, MCvFC, MCvMC, FSvFC, and SvC) and their overlaps, featuring (a) differentially expressed genes (DEG), (b) genes related to differentially methylated regions (DMRs), and (c) target genes identified from differentially expressed microRNAs (DMiR) analysis.

Next, we investigated overlaps among the 144 492 DEG, 37 382 DMR, and 352 DMiR counts (Figure 4). Overlaps between the MSvFS and MCvFC contrasts were found across all omic levels (412 DEG, 430 DMR, and 3 DMiR; Figure 4a–c). We then compared the four sex‐related contrasts against the SvC to identify overlaps that could be of particular relevance a sex‐specific perspective. The FSvFC contrast revealed the highest number of overlaps with SvC, suggesting that females are more responsive to stress stimuli across all omic levels (194 DEG, 287 DMR, and 6 DMiR).

Furthermore, the FSvFC contrast exhibited more DMR overlaps with the MSvFS contrast (*N* = 122) than with the MCvFC contrast (*N* = 59), indicating enhanced stress sensitivity in females at the epigenomic level. Conversely, at the transcriptomic level, an inverse relationship was observed, with FSvFC showing more overlaps with MCvFC (*N* = 629) than with MSvFS (*N* = 209). A higher number of overlaps was observed between MSvMC and MSvFS (*N* = 59) compared to MSvMC and MCvFC (*N* = 37). In contrast, transcriptomic differences were more pronounced, with MSvMC overlapping with MSvFS in 92 features and with MCvFC in 359 features. These findings show that stress tends to amplify sexual divergence at the epigenomic level while diminishing it at the transcriptomic level. Due to the relatively small number of DMiRs, making similar analyses at the miRNA level was not feasible.

**Figure 5:**
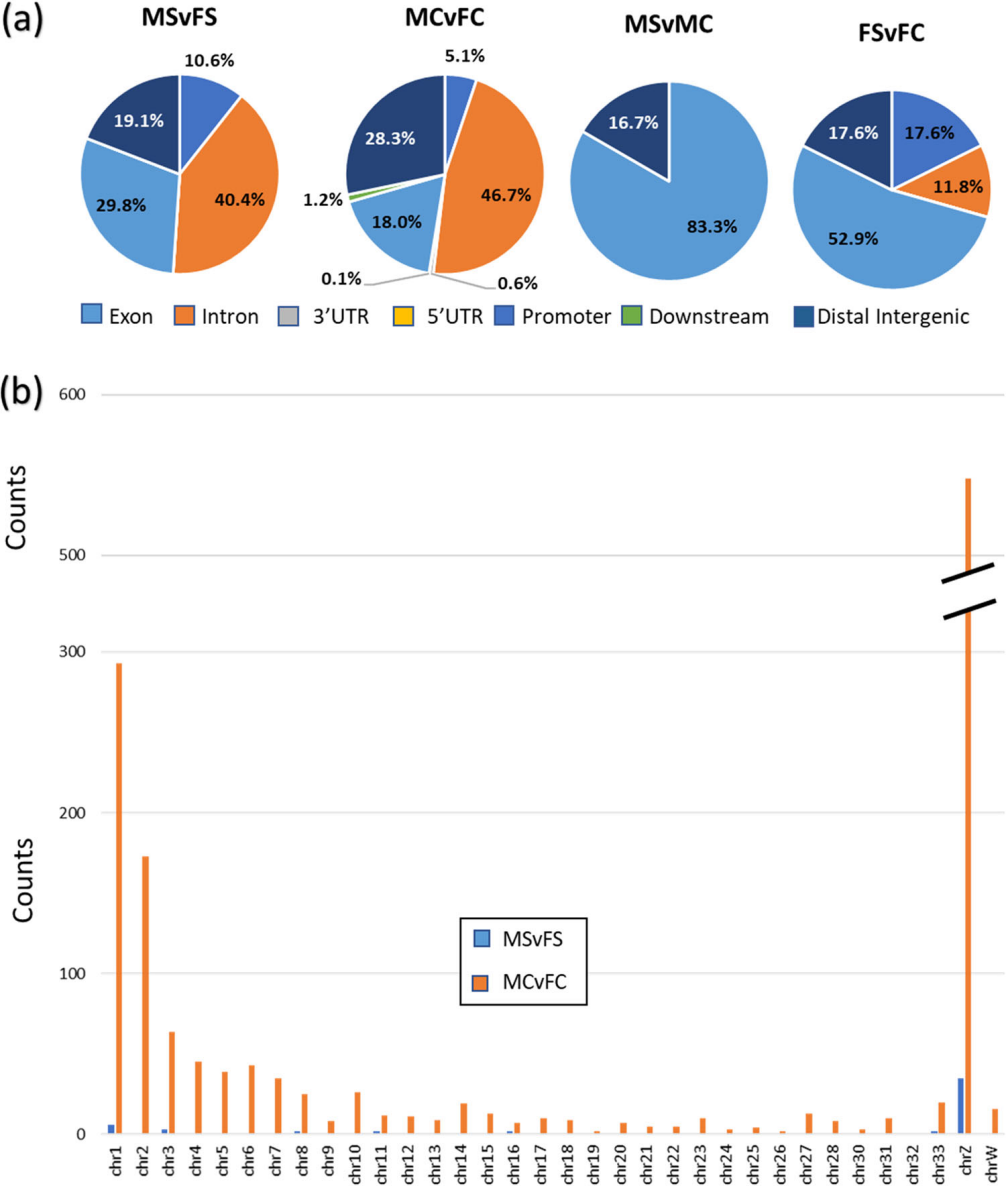
Annotation of DMRs showcases their connection to gene regions, such as intronic, exonic, promoter, and distal areas (a), and their distribution across chromosomes, with a focus on the Z chromosome (b), across the various contrasts explored in this research.

Annotation of the DMRs obtained against the chicken reference genome revealed 47 gene‐related DMRs for the MSvFS contrast, 1284 for MCvFC, 17 for FSvFC, and 6 for MSvMC (FDR ≤ 0.2; Supporting Information S1: Spreadsheet S1). A predominance of DMRs was observed in promoter regions, particularly in the contrasts within sexes (MSvMC and FSvFC), followed by intronic regions, mainly in the contrasts between sexes (SMvSF and CMvCF) (Figure 5a). A lower prevalence of DMRs was found in intergenic and exonic regions. Notably, most of the gene‐related DMRs identified in the male contrast (MSvMC) were concentrated in promoter regions, whereas in the female contrast (FSvFC) there was a substantial increase in exonic and intronic regions (Figure 5a). Additionally, the majority of the gene‐related DMRs in the MSvFS contrast showed an increase in exonic and promoter regions compared to MCvFC (Figure 5a). The number of gene‐related DMRs is substantially reduced in the MSvFS group compared to the MCvFC, with DMRs predominantly located on the Z chromosome (Figure 5b). Interestingly, no gene‐related DMR was identified in the FSvFC groups, while only one DMR was identified on the Z chromosome in the MCvMC contrast, mapped to the first intron of a novel gene (ENSGALG00000050678, GRCg6a). These observations led us to estimate the expectancy of DMRs in the Z chromosome relative to the total observed across all chromosomes, based on its genomic proportion (~0.65%) (Supporting Information S1: Spreadsheet S1). Interestingly, a higher than expected number of DMRs was observed on the Z chromosome for both the MSvFS (115) and MCvFC (65) contrasts.

We then investigated potential unbalancing/dose compensation on the Z chromosome, which differs in copy number between sexes (ZZ in males and ZW in females), potentially leading to imbalances in epigenetic regulation. To determine whether methylation patterns on the Z chromosome showed signs of dose compensation or sex‐biased imbalance, we normalized the Z chromosome data separately from the autosomal chromosomes and compared the *p*‐values before and after normalization. This normalization approach ensures that the observed differences are not artifacts of copy number differences, but instead reflect true biological or treatment‐induced changes. The observed correlations of 0.8 for MSvFS and 0.9 for MCvFC (*p* < 2.2e−16; Supporting Information S8: Figure S2) show that, in our data set, the methylation patterns on the Z‐chromosome are balanced between males and females.

**Figure 6:**
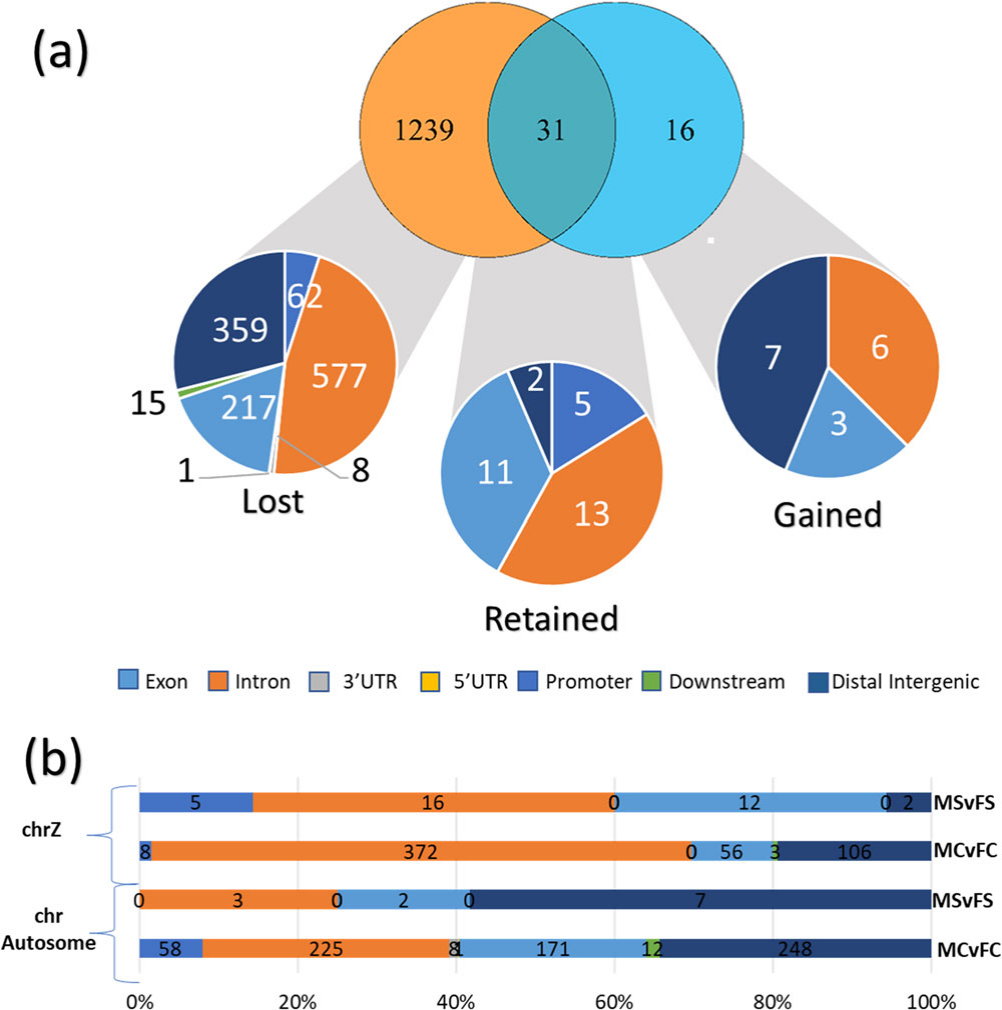
Venn diagram comparing DMR counts between sexes exposure to long‐term stress and annotation against the chicken reference genome of lost (left), retained (center), and gained (right) DMRs between sex of the stressed group in relation to the control group (a). Annotation of control and stressed DMRs relative to chicken genes, considering chromosomal location (b).

We then investigated the dynamics of DMRs related to sex. The comparison between MCvFC and MSvFS contrasts (Figure 6a) revealed that the majority of sex‐related DMRs in the control group are “lost” in the stress group (1239 DMRs), while only 33 DMRs were “retained” between both groups, and 16 DMRs were present only (“gained”) in the stress group (Supporting Information S2: Spreadsheet S2). The retained DMRs were primarily located in intronic regions (41.9%), followed by promoter (35.5%) and exonic regions (16.1%), and only 6.45% in intergenic regions. In contrast, the lost DMRs were predominantly found in intronic regions (46.6%), with intergenic (29.0%) and promoter regions (17.5%) also affected. Lastly, the gained DMRs were mainly located in intergenic regions (43.7%), followed by intronic (37.5%) and promoter regions (18.7%) (Figure 6a). Moreover, the distribution of sex‐related DMRs varied by chromosomal location: DMRs on both the Z sex chromosome and autosomes were primarily located in intronic and intergenic regions, followed by promoter areas. A similar pattern was observed for autosomal chromosomes in the stressed group. However, in the stressed group, a notable shift was observed, with a higher proportion of DMRs in promoter regions compared to intergenic regions (Figure 6b).

**Figure 7:**
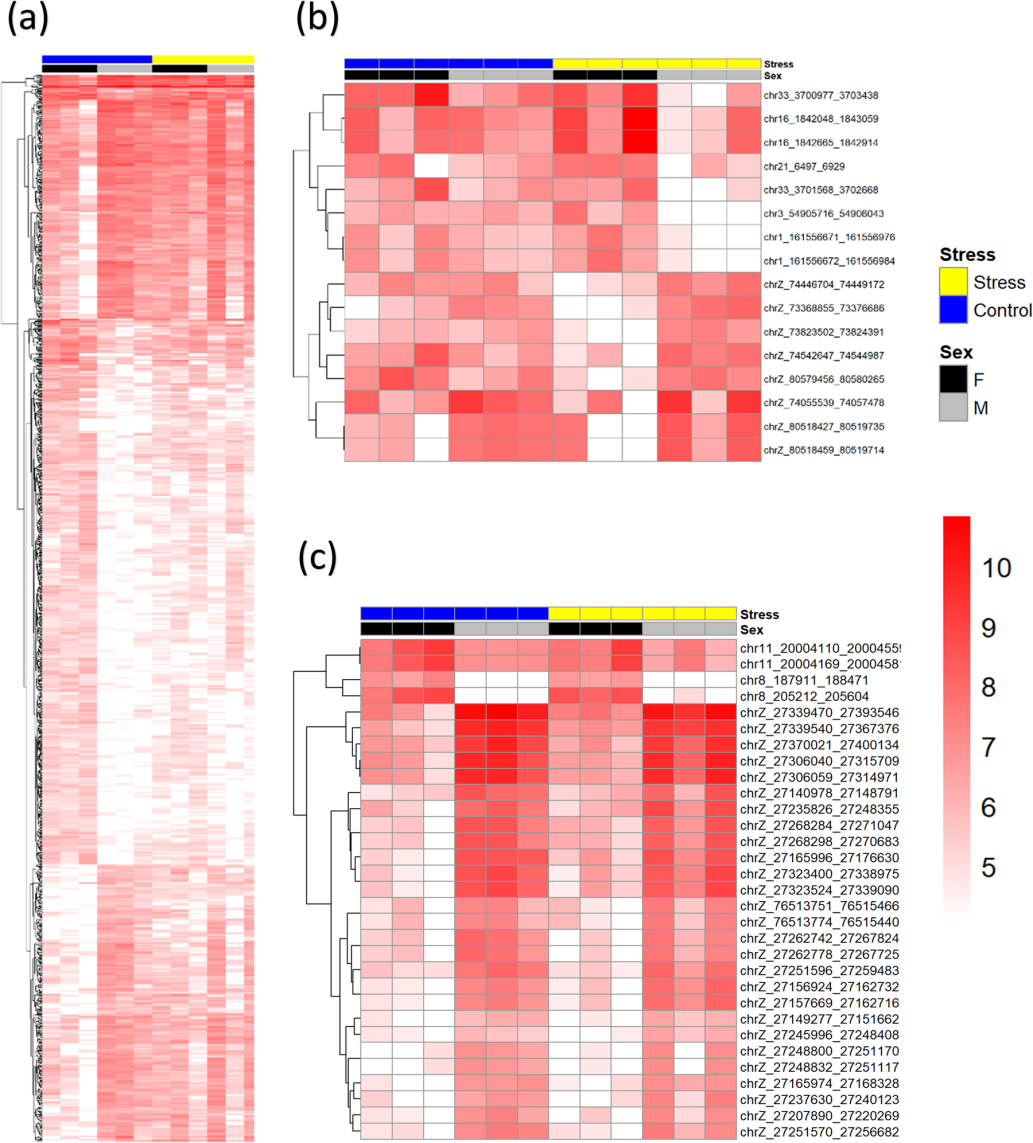
Heatmaps showing DMRs categorized as loss (a), gain (b), and remnants (c) between control (blue) and stress (yellow) conditions, stratified by sex (black: female; gray: male), with red intensity indicating methylation levels.

Building on the results shown in Figure 6a, we investigated the methylation levels of the identified “lost,” “gained,” and “retained” DMRs for male and female individuals using heatmaps (Figure 7). These heatmaps clearly demonstrate a loss of sexual dimorphism in methylation, driven by changes in the female stress group (Figure 7a). Additionally, while gained DMRs were fewer in number (Figure 7b), a distinct pattern emerged: on autosomal chromosomes, females exhibited hypermethylation relative to males, whereas, on the Z chromosome, males showed higher methylation levels. Finally, for the retained DMRs, the same pattern was observed: males exhibited hypermethylation on the Z chromosome, while females were hypermethylated on autosomal chromosomes (Figure 7c).

### 3.5 Omic Differences

**Figure 8:**
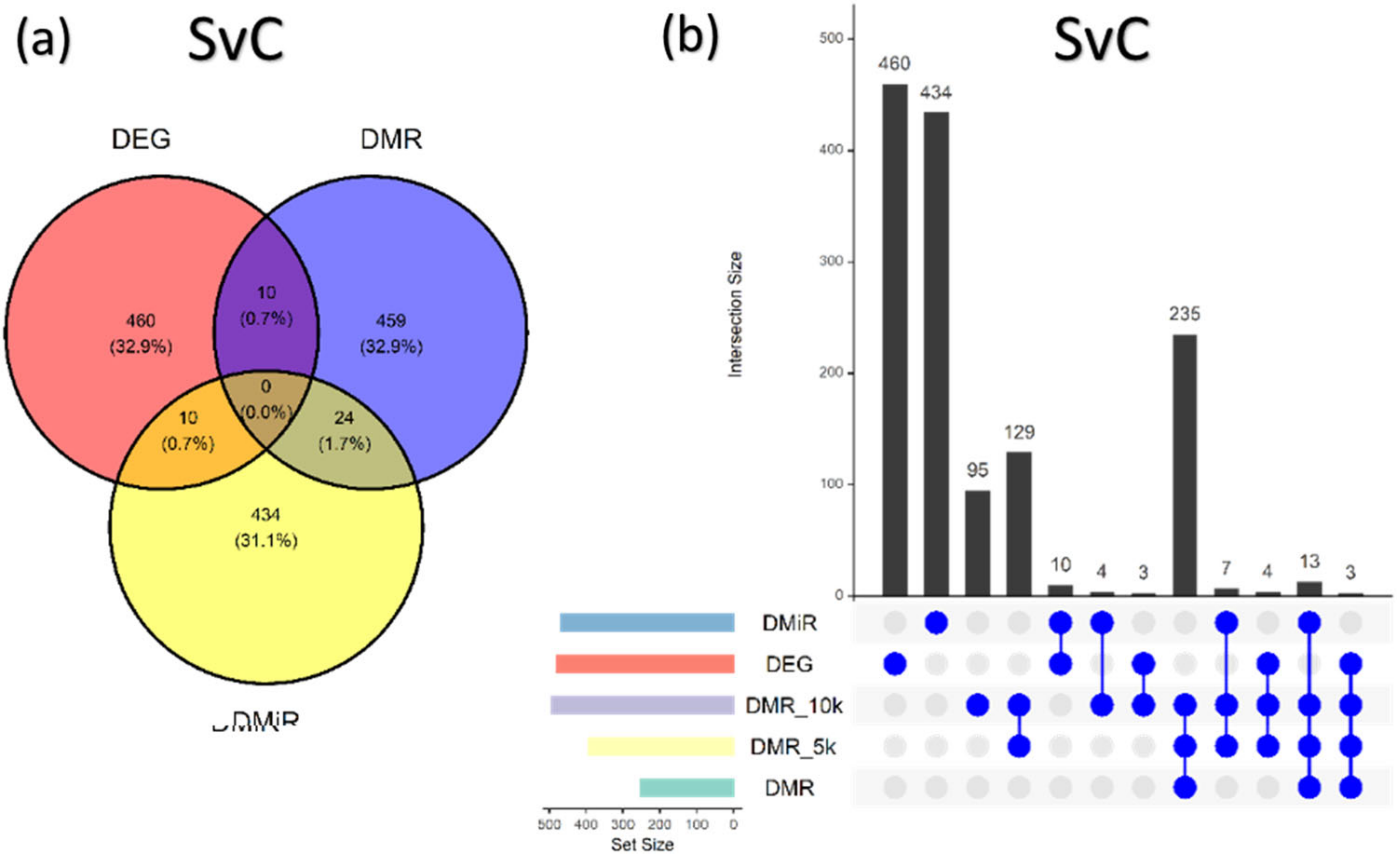
Venn diagram illustrating the distribution of omic features, differentiating DMiRs, DEGs, and DMRs, across the sex fixed model (SvC). The accompanying bar plot categorizes genomic features based on the position of DMRs relative to their nearest annotated gene, highlighting their distribution alongside DMiRs and DEGs.

The analysis of gene associations across the three omics layers—DEG, DMR, and DMiR—revealed a substantial proportion of these DMRs located within gene bodies. To further explore the genomic distribution of these DMRs, we examined their location relative to gene boundaries, by categorizing them into regions within genes (DMR), within ± 5 kb (DMR_5k), and within ± 10 kb (DMR_10k) of genes (Figure 8). A detailed examination of sex‐related differences is presented separately in Supporting Information S9: Figure S3.

Next, we analyzed the genes and genomic features associated with the omic changes found in the SvC contrast. The 534 significant DMRs identified encompassed 493 unique genes located within 10 kb of each DMR. Among these, 251 genes (51%) directly overlapped with significant DMRs, 140 (28%) were located within 5 kb, and 102 (21%) were within 10 kb of the significant DMRs (Figure 8b excludes “novel genes”; see Supporting Information S3: Spreadsheet S3). Most DMRs within genes were located in intronic regions (48.84%), followed by the promoter (28.05%), exonic (18.15%), and 3′ and 5′ UTR regions (4.95%).

**Figure 9:**
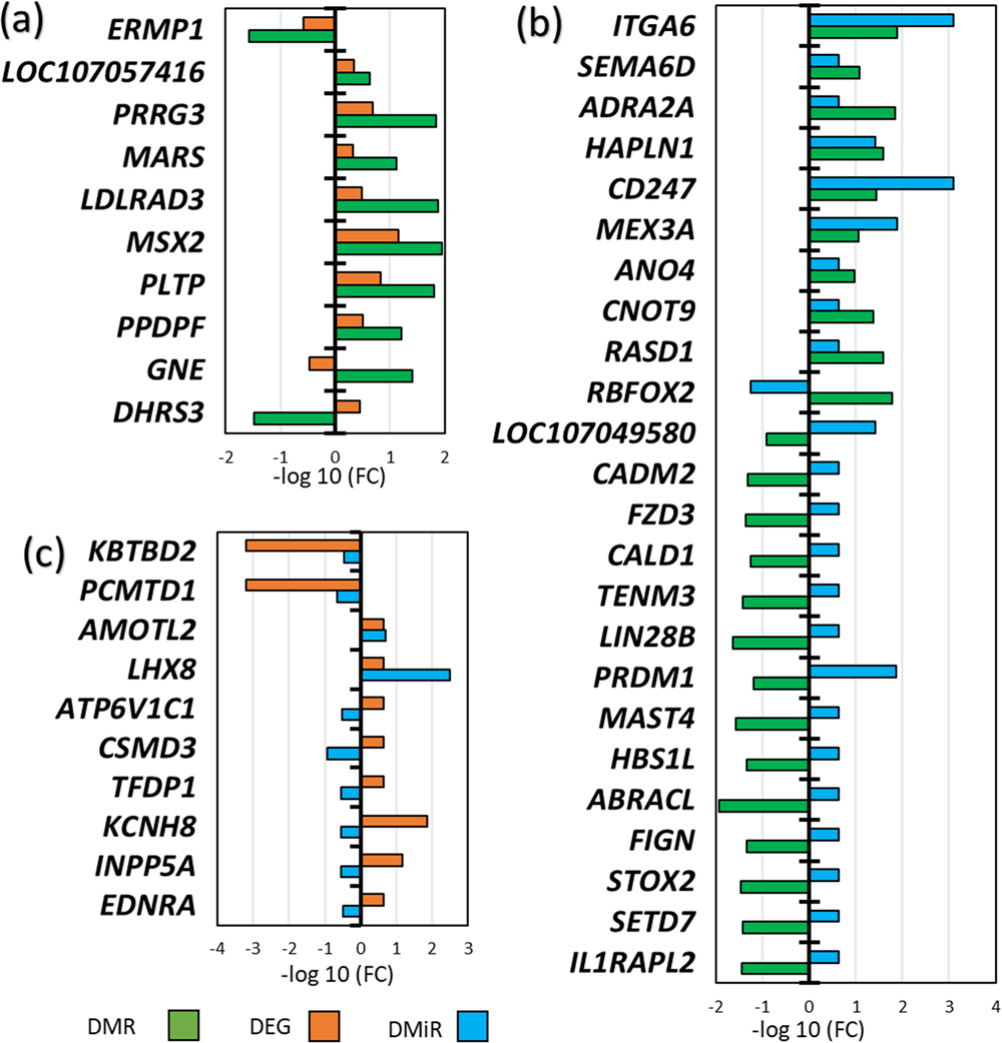
Relative levels and direction of significant omic changes in genes containing modifications in two omic levels: (a) DEG‐DMR, (b) DMR‐DMiR, and (c) DEG‐DMiR. Positive fold changes indicate that the transcriptome (DEG), miRNA (DMiR), or DMR‐related gene is higher expressed or hypermethylated in the stress group compared to the control group.

Following this, we aimed at uncovering potentially relevant molecular interactions among the omic changes observed that could affect genes of interest for the illumination stress response. For this, we focused on the 44 genes (10 DEG&DMR; 10 DEG&DMiR, and 24 DMiR&DMR) that contained significant changes (*p* < 0.05) in at least two of the three omic levels investigated in the SvC contrast (Figure 8a, intersection circles). Then, we retrieved the fold changes of these genes to identify the respective direction of the changes (Figure 9).

In most affected genes, hypermethylation was associated with increased gene expression (Figure 9a), with few exceptions, such as *ERMP1* and *DHRS3* (Table 2). Additionally, we identified 24 DMR‐DMiR intersections (Supporting Information S4: Spreadsheet S4), in which 15 (62.5%) hypomethylated DMRs were associated with increased miRNA expression, while 9 (37.5%) hypermethylated DMRs were associated with increased miRNA expression (Figure 9b). Inverse correlations were mostly observed for DMRs located closest to the gene, primarily in intronic regions (Table 2). We also identified DEG‐DMiR intersections, in which six genes exhibited an increase in gene expression associated with a decrease of DMiR expression (*LHX8, ATP6V1C1*, *CSMD3*, *TFDP1*, *KCNH8*, *INPP5A*, *EDNRA*), while the other four showed a positive relationship: two with increased expression (*AMOTL2* and *LHX8*) and two with decrease expression (*KBTBD2* and *PCMTD1*) (Figure 9c and Table 2).

**Table 2:** DMR location concerning omic intersections (DEG‐DMR and DMR‐DMiR).

Intersection	Ensembl‐ID	Symbol	DMR	DMR location in regard to the gene	DMiR	
			Loc‐id		ID	Pred‐score (%)
DMR‐DEG	ENSGALG00000030376	*DHRS3*	chr21:5353437‐5353707	5k–10k		
	ENSGALG00000015331	*GNE*	chrZ:52181936‐52183451	TSS‐5k		
	ENSGALG00000005849	*PPDPF*	chr20:9026483‐9028106	TSS‐5k		
	ENSGALG00000036686	*PLTP*	chr20:10545085‐10545389	5k–10k		
	ENSGALG00000038848	*MSX2*	chr13:10520320‐10521703	TSS‐5k		
	ENSGALG00000038913	*LDLRAD3*	chr5:19520281‐19520555	Intron		
	ENSGALG00000039315	*MARS*	chr33:4115446‐4116667	5k–10k		
	ENSGALG00000046724	*PRRG3*	chr4:10928685‐10929327	Promoter		
	ENSGALG00000049015	*LOC107057416*	chr23:104871‐107102	TSS‐5k		
	ENSGALG00000000438	*ERMP1*	chrZ:27782028‐27782591 chrZ:27782052‐27782545	Exon		
DMR‐DMiR	ENSGALG00000008888	*IL1RAPL2*	chr4:17142339‐17142993	Intron	gga‐miR‐30d	95.8
	ENSGALG00000009800	*SETD7*	chr4:29344636‐29345122 chr4:29344680‐29345009 chr4:29344725‐29344992	TSS‐5k	gga‐miR‐30d	91.0
	ENSGALG00000010646	*STOX2*	chr4:39687177‐39687388	Intron	gga‐miR‐30d	93.1
	ENSGALG00000011069	*FIGN*	chr7:20560989‐20561343	Intron	gga‐miR‐30d	86.1
	ENSGALG00000013826	*ABRACL*	chr3:54186198‐54186479	5k–10k	gga‐miR‐30d	95.6
	ENSGALG00000013962	*HBS1L*	chr3:55880443‐55880902	3′ UTR	gga‐miR‐30d	93.4
	ENSGALG00000014784	*MAST4*	chrZ:21082060‐21083236	Intron	gga‐miR‐30d	98.4
	ENSGALG00000015388	*PRDM1*	chr3:68346720‐68347900	TSS‐5k	gga‐miR‐219a	92.7
	ENSGALG00000026761	*LIN28B*	chr3:68894555‐68894909	5k–10k	gga‐miR‐30d	99.0
	ENSGALG00000030065	*TENM3*	chr4:40449569‐40449953	Intron	gga‐miR‐30d	92.2
	ENSGALG00000033471	*CALD1*	chr1:62432659‐62432956	Promoter	gga‐miR‐30d	95.3
	ENSGALG00000042308	*FZD3*	chr3:105830604‐105830941	TSS‐5k	gga‐miR‐30d	90.9
	ENSGALG00000043393	*CADM2*	chr1:94775199‐94775607	Intron	gga‐miR‐30d	91.1
	ENSGALG00000054703	*LOC107049580*	chr25:1884872‐1885840	TSS‐5k	gga‐miR‐6649‐5p	100
	ENSGALG00000012540	*RBFOX2*	chr1:51933809‐51934173	Intron	gga‐miR‐7467‐3p	94.7
	ENSGALG00000004860	*RASD1*	chr14:5189030‐5191178	3′ UTR	gga‐miR‐30d	95.9
	ENSGALG00000011402	*CNOT9*	chr7:22482700‐22483735	5k–10k	gga‐miR‐30d	96.4
	ENSGALG00000011614	*ANO4*	chr1:47530079‐47530522	Intron	gga‐miR‐30d	82.8
	ENSGALG00000014016	*MEX3A*	chr25:738520‐738986	3′ UTR	gga‐miR‐19a‐5p	91.7
	ENSGALG00000015441	*CD247*	chr1:93069721‐93070726	TSS‐5k	gga‐miR‐6612‐5p	93.9
	ENSGALG00000015627	*HAPLN1*	chrZ:62780620‐62782062 chrZ:62802651_‐62804383 chrZ:62802662‐62803939	Intron	gga‐miR‐6649‐5p	91.2
	ENSGALG00000023521	*ADRA2A*	chr6:27201927‐27202295 chr6:27201950‐27202181	5k–10k	gga‐miR‐30d	97.2
	ENSGALG00000030542	*SEMA6D*	chr10:10366403‐10366757	Exon	gga‐miR‐30d	87.6
	ENSGALG00000034007	*ITGA6*	chr7:17712400‐17712612	TSS‐5k	gga‐miR‐6612‐5p	85.8

*Note:* Pred‐score is the prediction score for a gene to be targeted by a specific miRNA.

#### 3.5.1 Gene Enrichment and Functional Module Inference

We then performed functional module inference to gain deeper insights into the biological functions and interactions of the identified genes [62]. By using the *G. gallus* PPI network from STRING v.11 and filtering for interactions with a confidence score higher than 700, we identified 214 694 interactions across 8366 genes.

**Figure 10:**
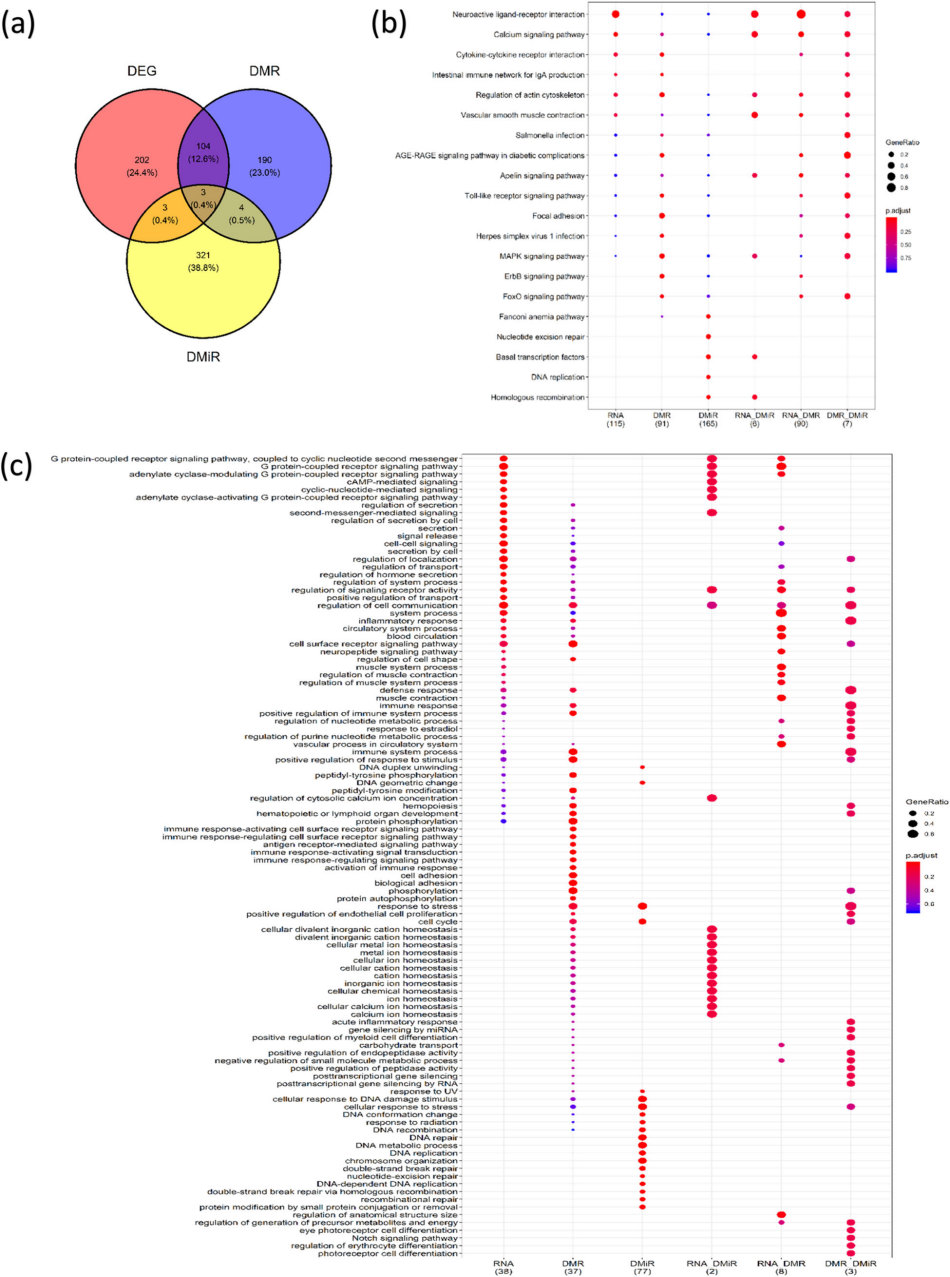
Venn diagram showing (a) unique and intersected genes among different omic level features (*p* < 0.05) obtained using SvC after the module approach,(b) gene enrichment using KEGG pathways, and (c) GO terms.

From this analysis, 114 intersected genes were identified (Figure 10a; intersection circles), which were used for KEGG (Figure 10b) and GO term (Figure 10c) gene enrichment (Supporting Information S5: Spreadsheet S5). Three genes (*GNG12*, *ADRA1B*, and *GRM5F*) overlapped across all three omic levels (Figure 10a). The KEGG pathway “Neuroactive ligand‐receptor interaction” was enriched for genes across all intersections (Figure 10b), while the GO terms “signaling molecules linked to G‐protein coupled receptors” and “cAMP‐mediated signaling” were enriched among different intersections (Figure 10c).

**Figure 11:**
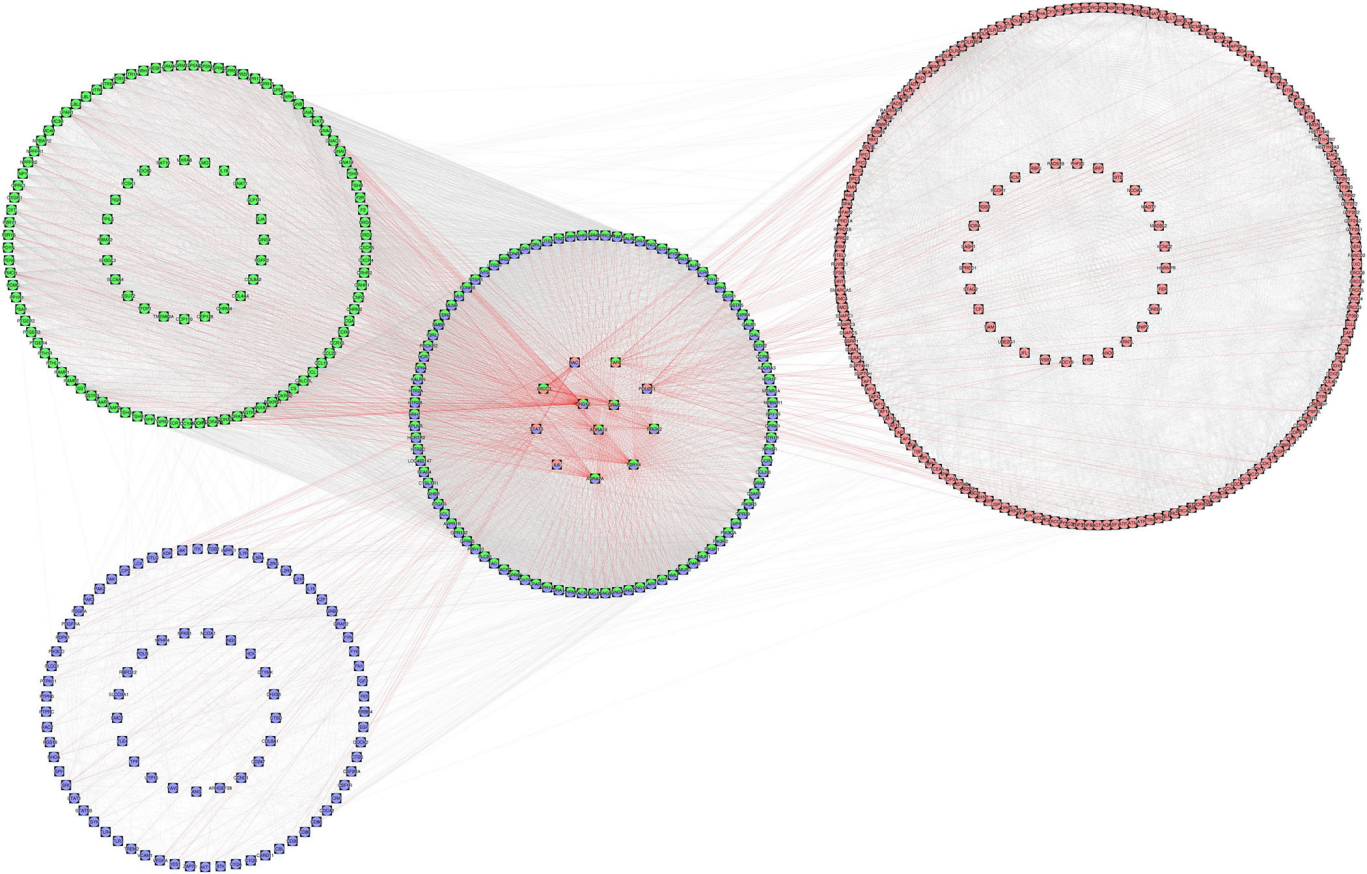
Gene–protein interaction network obtained from the module inference approach from DEG (green), DMR (blue), and DMiR (red) of all the inputted genes from the module approach. The inner circles represent the original genes from the three genomic levels, and the outer circles represent the genes that emerged from the module interaction inference. Red lines represent the interactions between the 12 core genes and other genes.

From the module inference approach, we identified 827 significant genes across the three omic levels analyzed (DEG, DMR, and DMiR). Of these, 544 genes were considered to have the highest confidence level of interaction (conf. > 90%). From this group, we selected 523 genes connected to the main module network for the final analysis (Figure 11). Among these 523 genes, 86 were part of the original data set from DEG, DMR, and/or DMiR, while 437 genes emerged from the module approach.

We then analyzed the interaction patterns of the genes that emerged through the module analysis from the original gene set (86 genes) and the 114 intersection genes across different omic levels (Figure 11; inner big circle). Most of the intersected genes resulted from the DEG‐DMR (green/blue) intersection, while the other two intersections were found only in the original data set. The 12 core genes (Figure 11, inner small circle) from the original data, which form the basis of these interactions, are: *ADRA1B, GRM5, GNG12* (DEG‐DMR‐DMiR)*; ADRA2A, P2RY4, PROK2* (DEG‐DMR)*; IL6, RAC1, STAT3, POU2F1* (DMR‐DMiR)*; and TAF8, XRCC3* (DEG‐DMiR).

After applying the module approach, we identified 190 genes associated exclusively with DMR, 202 genes linked to DEG, and 321 related to DMiR. Additionally, 104 genes exhibited interactions between DMR and DEG, while 4 genes were shared between DMR and DMiR, and another 3 genes were common across all three omic layers (DMR, DEG, DMiR) (Figure 11).

To better understand the function of these genes, we conducted pathway enrichment analysis, identifying significant biological pathways, including cAMP‐mediated signaling, leukocyte chemotaxis, and migration. A total of 188 genes were analyzed: 86 original genes (including the 12 core intersected genes) plus 102 intersected genes from the module analysis. These genes were grouped into clusters based on their biological functions, with particular emphasis on pathways related to the circadian cycle. For further details on the original omic data for the 12 intersected genes, please refer to Supporting Information S6: Spreadsheet S6.

**Figure 12:**
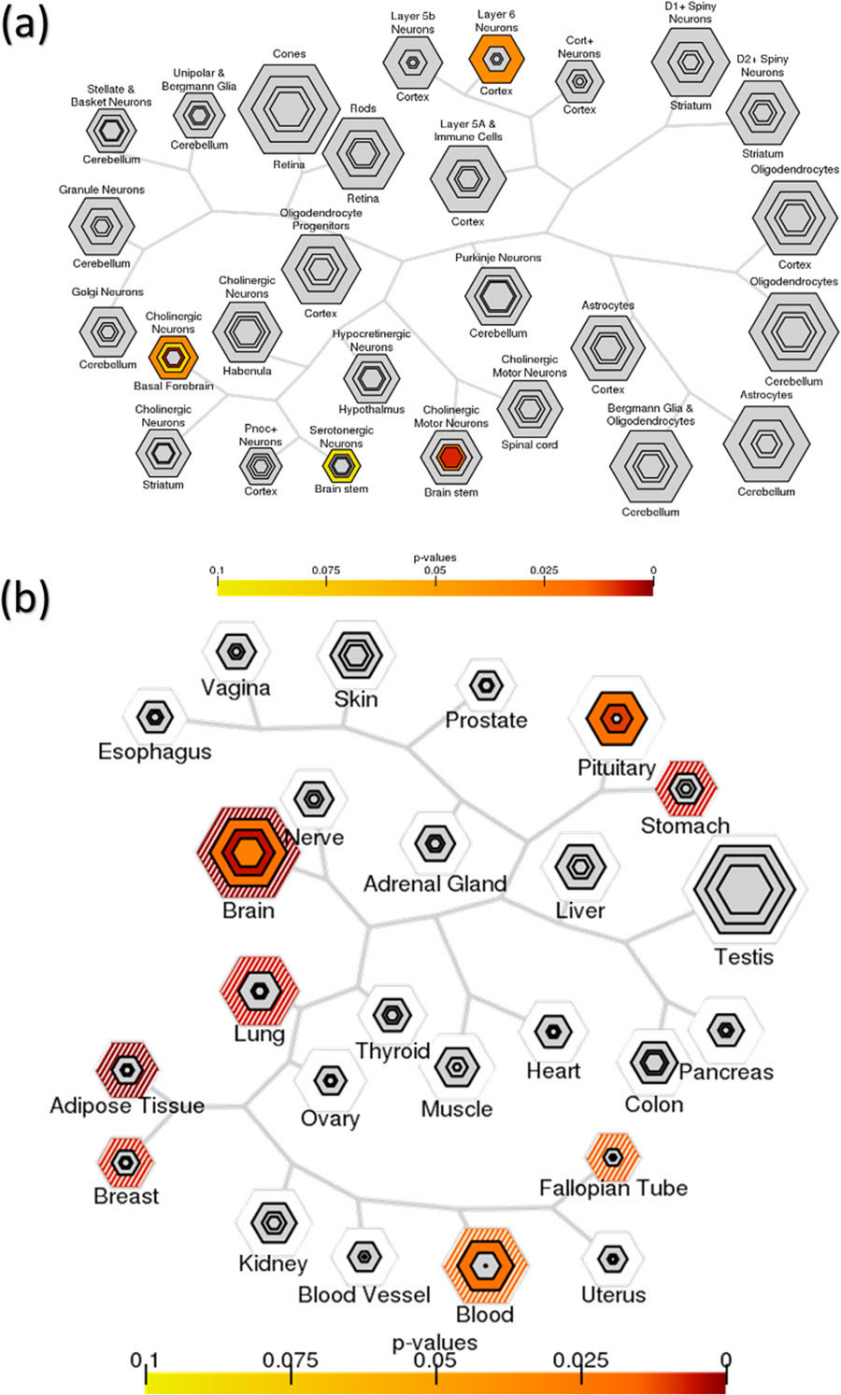
Tissue specific expression analysis (TSEA) (a) and cell‐type specific expression analysis (CSEA) (b) of the 114 intersected genes among different omic levels (*p* < 0.05), obtained using SvC after the module approach.

To verify the biological relevance of the 114 intersected genes to our study, we performed a tissue‐specific expression analysis (TSEA). In the brain regions, Layer 6 Neurons were enriched in the cortex, Cholinergic Neurons were enriched in the basal forebrain, and Serotonergic and Cholinergic Motor neurons were enriched in the brainstem (Figure 12a). Additionally, we conducted a cell‐type specific expression analysis (CSEA), which showed enrichment in the brain, pituitary, fallopian tube, blood, stomach, adipose tissue, and breast (Figure 12b).

## 4 Discussion

In the present study, we took an integrative multi‐omic approach to shed light on epigenetic and transcriptomic responses of the avian pineal gland to fluctuating illumination patterns, focusing on sex‐specific responses. For this, we exposed chickens to unpredictable illumination patterns during rearing and then performed an integrative analysis of DNA methylation, miRNA, and gene expression in the pineal gland to elucidate the molecular interplay among these different omic levels.

Numerous studies highlight the sex‐specific effects of stress, particularly its impact on brain function and development. Prenatal stress and early‐life manipulations have been shown to exacerbate developmental vulnerability to sex‐specific neuropsychiatric disorders in mice [63]. Sex‐dependent processes are a recurrent theme in prenatal stress literature, significantly influencing disease severity and onset [64]. For instance, posttraumatic stress disorder is known to contribute to sexual dysfunction in humans [65]. Prenatal stress also plays a crucial role in neural and behavioral sexual differentiation in rodents [66], impairing feminine‐typical copulatory behavior in female mice [67] and increasing masculine‐typical patterns of juvenile play and adult behavior in female guinea pigs [68]. In birds, transcriptomic and epigenetic responses to environmental stimuli, including light exposure, have demonstrated strong sex‐dependent patterns, particularly in the brain [33, 43]. These findings emphasize that environmental factors interact with genetic programming to shape brain function in a sex‐dependent manner.

In our study, multiple pieces of evidence demonstrate sex‐specific effects caused by stress, predominantly affecting females across various omic levels. First, in the MDS test, females clustered with the stress group, while males clustered with the control group for DEG and DMR genomic features. Second, illumination stress increased sex differences in gene expression, while decreasing sex‐specific methylomic differences. Third, the number of overlaps in the SvC contrast with other contrasts further highlighted these patterns. Fourth, there was a substantial DMR loss in females from the stress group compared to males. Taken together, these findings suggest that stress responses tend to increase sexual divergence—primarily affecting females—at the epigenomic level, while reducing sexual differences at the transcriptomic level. Moreover, the females subjected to stress tend to exhibit a pineal methylome more similar to that of males, suggesting that light‐induced stress disrupts the differentiation of the heterogametic sex and leads to a loss of sexual dimorphism at the epigenomic level. However, this pattern is not as pronounced at the transcriptomic level, where sex differences persist. Previous research has demonstrated that different sexes maintain distinct epigenomic profiles in the brain [69], but the mechanisms underlying this balance remain unclear.

Interestingly, a previous study from our group, which focused on the mitochondrial genome from the same experimental model, revealed that stress‐induced methylation changes were more pronounced in males [46]. Since mitochondria are exclusively inherited from the mother, these modifications could reflect a mechanism that results in balanced mito‐nuclear interactions across sexes. In this context, the observation that females under prolonged stress shift their nuclear pineal epigenome toward the male profile could represent a regulatory adjustment to align nuclear and mitochondrial contributions, ultimately stabilizing gene expression and preserving sexual dimorphism at the transcriptomic level.

In birds, the Z chromosome is not inactivated, and the heterogametic sex is female [70, 71], whereas in mammals, the male is heterogametic and the female is homogametic, with one of the X chromosomes inactivated [72]. Due to this, we would expect an overrepresentation of false‐positive DMRs showing hypermethylation in males. However, methylation on the Z chromosome was statistically equalized between males and females, concordant with previous findings showing that gene expression is equalized between sexes in birds [70]. Notably, DMR sex differences occurred more frequently than expected by chance on the Z chromosome in both the illumination stress and control groups, although the appearance above expectancy is even higher in the stress groups. Also, in the retained and gained DMRs in the stress group, there was a relative increase in epigenetic differences on the Z chromosome. Despite the substantial loss of DMRs between sexes in the stress group, nearly all DMRs on the Z chromosome were hypermethylated in males relative to females, while DMRs on autosomal chromosomes were hypermethylated in females compared to males. This pattern was observed both in the retained and gained DMRs.

Two of the gained DMRs were located in intronic regions of the *MHCBL2* gene, and one in the *IFNGR1* gene. Another DMR was located distal to the *KLHDC7A* gene. Interestingly, all three of these genes are related to immune system processes: *MHCBL2* is a class II histocompatibility antigen, essential for immune function [73], *IFNGR1* encodes the interferon gamma receptor 1, which is crucial for innate immune defense [74], and *KLHDC7A* is associated with diabetic retinopathy [75]. Of the 31 remaining DMRs, only 1 was located distal to a known gene, *LOC112532921*, which is activated by G‐protein‐coupled receptor‐mediated signal transduction and plays a key role in intracellular signal transduction (NCBI, GENEID: 5331).

DMR distribution differed between the stress and control groups, particularly in relation to chromosomal location (Figure 6a). DMRs retained in the stress group were predominantly located in intronic (41.9%), promoter (35.5%), and exonic (16.1%) regions, with minimal presence in intergenic regions (6.45%). In contrast, lost DMRs were more frequently found in intergenic (29.0%) and intronic (46.6%) regions, while gained DMRs were mainly intergenic (43.7%), followed by intronic (37.5%) and promoter (18.7%) regions. These patterns suggest that the illumination stress selectively alters regulatory elements while preserving functionally relevant methylation sites. When comparing autosomal and Z chromosomal DMRs (Figure 6b), autosomal DMRs in both groups were mostly intronic and intergenic, with the stress group showing an increased proportion of promoter‐associated DMRs. Conversely, on the Z chromosome, DMRs were enriched in intronic and promoter regions, with stress further increasing their presence in promoters over intergenic regions. This redistribution under stress suggests a regulatory shift, particularly in sex chromosomes. Since methylation plays a crucial role in gene regulation, its redistribution under stress may have functional consequences. It is well established that methylation in regulatory regions, such as promoters, is typically associated with transcriptional repression, while methylation within gene bodies is often linked to high levels of gene expression [76, 77, 78]. Thus, a deeper investigation into these retained and gained DMRs could provide valuable insights into diseases that arise from sex‐specific mechanisms in various animal species.

To gain insights into the relationship between the DMRs and DEGs generated by the illumination stress, we classified DMRs into three categories: those within genes, those between the TSS and 5 kb, and those between 5 and 10 kb of DEGs. Most of our DMRs were located within genes (Supporting Information S3: Spreadsheet S3). Previous research suggests that DMRs within genes, particularly between exons, splice sites, and flanking‐intronic regions, are involved in regulating alternative splicing [79]. We thus considered a 5 kb distance from the TSS as part of the regulatory region of these genes. On the other hand, most cell‐type‐specific DNA methylation occurs at distal regulatory elements, often overlapping with active enhancers [80, 81]. The regulation and functional consequences of DNA methylation heterogeneity in these regions remain unclear [82]. Enhancers, which are cis‐acting elements, can be located up to 10 kb from genes [83, 84], which is why we included this third category in our DMR classification.

Omic interactions are complex and largely dependent on where DMRs are located. Our results show that most highly expressed genes overlapped with hypermethylated DMRs or were directly correlated with gene expression. Positive correlations were predominantly found in DMRs located within or inside genes, while negative correlations were mostly associated with DMRs located distally. Furthermore, most of the DMRs closest to the genes were inversely correlated with target genes from differential miRNA expression.

Gene enrichment analysis helps identify collective biological functions by systematically mapping genes and proteins to their associated biological annotations [85]. We performed functional module inference to integrate gene expression networks and identify genes interacting with proteins and other molecules [62]. Genes intersecting among the three omic levels were identified and then used for KEGG and GO term enrichments. These 114 genes displayed strong interactions (confidence > 90%) and enriched pathways related to the nervous and immune systems, G‐protein coupled receptors, and cAMP‐mediated signaling. The Neuroactive ligand‐receptor interaction KEGG pathway was enriched across all intersections, as were signaling molecules linked to G‐protein coupled receptors, both previously linked to circadian rhythm disruption [86, 87]. Additionally, cAMP‐mediated signaling GO terms were enriched among the intersections. This pathway is known to regulate circadian rhythm via G‐protein coupled receptors, affecting the phase and period of the clock [88]. Moreover, cAMP plays a role in melatonin synthesis, which is crucial for circadian regulation [89, 90].

From the 114 intersection genes identified through the module inference approach, 12 were highlighted in our previous analysis (DMR, DMiR, and/or DEG). However, they became intersection genes due to their interaction with proteins. Most of these genes were originally identified as DMiRs, with *ADRA2A* and *PROK2* identified as DMRs, and *P2RY4*, *TAF8*, and *XRCC3* identified as DEGs. These 12 genes are all directly or indirectly related to the circadian cycle. ***PER2*** is one of the nine master circadian clock genes located in the SCN and has been suggested as a tumor suppressor [91]. ***PO2F1*** encodes a transcription factor involved in immune modulation. When its activity is increased, it interacts with *PER2*, leading to a protumorigenic effect [91]. ***PROC2*** encodes a protein expressed in the SCN, which may function as an output component of the circadian clock in humans [92, 93]. ***STAT3*** is highly expressed during the day in the SCN [94], playing a role in the circadian clock's response to systemic inflammatory stimuli and being involved in immune responses [94], particularly in the IL6‐mediated induction of target genes [95]. IL‐6, a pleiotropic cytokine, has been proposed as a “sleep factor,” with its circadian secretion correlating with sleep/sleepiness [85]. ***ADRA2A*** is an adrenergic receptor that plays a critical role in regulating neurotransmitter release from sympathetic nerves and adrenergic neurons in the central nervous system [96]. ***RAC1*** encodes a G protein involved in efferocytosis, a process regulated by the circadian rhythm and essential for tissue homeostasis [97]. ***P2RY4*** encodes the P2Y purinoceptor 4, part of the G‐protein coupled receptor family (Gene ID: 5030, NCBI). Purinergic signaling via P2Y4 plays a role in circadian regulation, as extracellular ATP oscillations help synchronize internal clocks with environmental cues [98]. ***TAF8*** is a TATA‐box binding protein‐associated factor, and ***XRCC3*** participates in homologous recombination, helping to maintain chromosome stability and repair DNA damage [99]. These two genes were annotated in the Circadian Gene Database (http://cgdb.biocuckoo.org/) as being differentially expressed in studies challenging the circadian cycle. The involvement of *TAF8* and *XRCC3* in transcription and DNA repair suggests a role in maintaining circadian homeostasis under stress. Given the observed nuclear (present data) and mitochondrial [46] epigenetic shifts, *XRCC3* may contribute to genomic stability, helping preserve transcriptomic sex differences despite underlying methylomic changes.

Importantly, three genes—*GNG12, ADRA1B*, and *GRM5*—overlapped across the three omic levels and are part of the Circadian Gene Database. *GNG12* is a G‐protein, while *ADRA1B* is an adrenergic receptor that regulates cell growth and proliferation. This gene was considered a top circadian gene in a study about aging effects on circadian patterns in the human prefrontal cortex [100]. *GRM5* is a glutamate metabotropic receptor shown to regulate neural network activity and synaptic plasticity [101]. These G‐protein coupled receptors are known to regulate the molecular oscillator and the phase/period of the clock [88].

Following this, we performed a TSEA of the 114 intersection genes, which revealed enrichment in specific brain regions, including Layer 6 neurons, cholinergic neurons in the basal forebrain, and serotonergic motor neurons in the brainstem. While the cerebellar cortex oscillator has been demonstrated through clock gene expression within cerebellar layers [102], the cholinergic forebrain arousal system is shown to act directly on the circadian pacemaker [103]. The cholinergic system helps the circadian system to maintain a 24‐h cycle [104].

Finally, CSEA showed enrichment in the brain, pituitary, fallopian tube, blood, stomach, adipose tissue, and breast (Figure 12b). SCN neurons in birds can synchronize circadian clocks in peripheral tissues such as the lungs, heart, liver, and pineal gland [105]. This analysis strongly suggests that these genes are predominantly expressed in hypothalamic–pituitary–thyroid axis‐related tissues [106], which is a key regulator of the circadian cycle.

### 4.1 Final Considerations

This study reveals how three levels of omics interact to respond to environmental factors, driving sex‐specific metabolic and physiological changes in the pineal gland of chickens subjected to long‐term Illumination stress.

Our multi‐omic analysis provides a consistent narrative of the orchestral interaction of genes in response to prolonged environmental exposure. We suggest this “orchestra” is led by 12 key genes related to the circadian cycle, which interact strongly with 102 secondary genes identified through the integration of three distinct omic levels. These genes, in turn, interact with many others, providing a detailed map of the molecular responses of the pineal gland to illumination stress.

Furthermore, our findings show the loss of sex‐specific epigenetic differences under illumination stress, driven predominantly by female responses. This suggests that the heterozygotic sex might be the most sensitive, potentially shifting their epigenetic profile toward a male‐like state. These findings provide a framework for exploring how environmental stressors interact with sex differentiation and epigenetic regulation, offering broader insights into circadian stress response across species. Our findings open new avenues for researchers interested in understanding sex‐specific disruptions in circadian regulation, with implications for health and also animal welfare.

## Author Contributions

Conceptualization: Carlos Guerrero‐Bosagna, Per Jensen, and Fábio Pértille. Methodology and sample collection: Carlos Guerrero‐Bosagna, Nina Mitheiss and Pia Løtvedt. Biological Resources: Per Jensen. Data analysis: Fábio Pértille, Tejaswi Badam, and Carlos Guerrero‐Bosagna. Writing – original draft: Fábio Pértille, Carlos Guerrero‐Bosagna, and Nina Mitheiss. Writing – review and editing: Carlos Guerrero‐Bosagna, Fábio Pértille, Luiz Lehmann Coutinho, Tejaswi Badam, Per Jensen, Mika Gustafsson, Pia Løtvedt, Emmanouil Tsakoumis, and Nina Mitheiss. Funding acquisition: Per Jensen and Carlos Guerrero‐Bosagna. All the authors read and agree with the latest version of the manuscript.

## Conflicts of Interest

The authors declare no conflicts of interest.

## Supporting information

Supporting information.

Supporting information.

Supporting information.

Supporting information.

Supporting information.

Supporting information.

Supporting information.

Supporting information.

Supporting information.

Supporting information.

Supporting information.

## Data Availability

The data set supporting the conclusions of this article is available from the European Nucleotide Archive (ENA) repository (EMBL‐EBI), under accession number PRJEB35831 (https://www.ebi.ac.uk/ena/browser/view/PRJEB35831). All the programs and R packages used in this protocol are open access and all the scripts are available at github online (https://github.com/fpertille/PinealGlandChicken).

## References

[1] K. N. Morgan and C. T. Tromborg , “Sources of Stress in Captivity,” Applied Animal Behaviour Science 102, no. 3–4 (February 2007): 262–302.

[2] L. Frésard , M. Morisson , J. M. Brun , et al., “Epigenetics and Phenotypic Variability: Some Interesting Insights From Birds,” Genetics, Selection, Evolution 45, no. 1 (2013): 16. 10.1186/1297-9686-45-16 PMC3693910 23758635

[3] S. Shini , A. Shini , and G. R. Huff , “Effects of Chronic and Repeated Corticosterone Administration in Rearing Chickens on Physiology, the Onset of Lay and Egg Production of Hens,” Physiology & Behavior 98, no. 1–2 (August 2009): 73–77. 19393674 10.1016/j.physbeh.2009.04.012

[4] C. Nicol , The Behavioural Biology of Chickens (Wallingford, 2015).

[5] K. Ikegami and T. Yoshimura , “Comparative Analysis Reveals the Underlying Mechanism of Vertebrate Seasonal Reproduction,” General and Comparative Endocrinology 227 (February 2016): 64–68. 26050562 10.1016/j.ygcen.2015.05.009

[6] R. Orozco‐Solis and P. Sassone‐Corsi , “Epigenetic Control and the Circadian Clock: Linking Metabolism to Neuronal Responses,” Neuroscience 264 (April 2014): 76–87. 24486964 10.1016/j.neuroscience.2014.01.043 PMC6501790

[7] A. Shankar and C. T. Williams , “The Darkness and the Light: Diurnal Rodent Models for Seasonal Affective Disorder,” Disease Models & Mechanisms 14, no. 1 (January 2021): dmm047217, 10.1242/dmm.047217. 33735098 PMC7859703

[8] M. M. Macchi and J. N. Bruce , “Human Pineal Physiology and Functional Significance of Melatonin,” Frontiers in Neuroendocrinology 25, no. 3–4 (September 2004): 177–195. 15589268 10.1016/j.yfrne.2004.08.001

[9] K. L. Gamble , R. Berry , S. J. Frank , and M. E. Young , “Circadian Clock Control of Endocrine Factors,” Nature Reviews Endocrinology 10, no. 8 (August 2014): 466–475. 10.1038/nrendo.2014.78 PMC4304769 24863387

[10] P. F. Devlin and S. A. Kay , “Circadian Photoperception,” Annual Review of Physiology 63, no. 1 (March 2001): 677–694. 10.1146/annurev.physiol.63.1.677 11181972

[11] J. F. Duffy and K. P. Wright , “Entrainment of the Human Circadian System by Light,” Journal of Biological Rhythms 20, no. 4 (August 2005): 326–338. 16077152 10.1177/0748730405277983

[12] J. M. McGoogan and V. M. Cassone , “Circadian Regulation of Chick Electroretinogram: Effects of Pinealectomy and Exogenous Melatonin,” American Journal of Physiology‐Regulatory, Integrative and Comparative Physiology 277, no. 5 (November 1999): R1418–R1427. 10.1152/ajpregu.1999.277.5.R1418 10564215

[13] A. B. Dollins , I. V. Zhdanova , R. J. Wurtman , H. J. Lynch , and M. H. Deng , “Effect of Inducing Nocturnal Serum Melatonin Concentrations in Daytime on Sleep, Mood, Body Temperature, and Performance,” Proceedings of the National Academy of Sciences 91, no. 5 (March 1994): 1824–1828. 10.1073/pnas.91.5.1824 PMC43256 8127888

[14] V. M. Cassone and D. F. Westneat , “The Bird of Time: Cognition and the Avian Biological Clock,” Frontiers in Molecular Neuroscience 5 (2012): 32, 10.3389/fnmol.2012.00032. 22461765 PMC3309970

[15] S. P. Karaganis , P. A. Bartell , V. R. Shende , A. F. Moore , and V. M. Cassone , “Modulation of Metabolic and Clock Gene mRNA Rhythms by Pineal and Retinal Circadian Oscillators,” General and Comparative Endocrinology 161, no. 2 (April 2009): 179–192. 19136000 10.1016/j.ygcen.2008.12.015 PMC2728004

[16] D. Bell‐Pedersen , V. M. Cassone , D. J. Earnest , et al., “Circadian Rhythms From Multiple Oscillators: Lessons From Diverse Organisms,” Nature Reviews Genetics 6, no. 7 (July 2005): 544–556. 10.1038/nrg1633 PMC2735866 15951747

[17] S. P. Karaganis , V. Kumar , P. D. Beremand , M. J. Bailey , T. L. Thomas , and V. M. Cassone , “Circadian Genomics of the Chick Pineal Gland In Vitro,” BMC Genomics 9, no. 1 (2008): 206. 18454867 10.1186/1471-2164-9-206 PMC2405806

[18] S. Gaston and M. Menaker , “Pineal Function: The Biological Clock in the Sparrow?,” Science (1979) 160, no. 3832 (June 1968): 1125–1127. 10.1126/science.160.3832.1125 5647435

[19] M. Zeman and I. Herichová , “Circadian Melatonin Production Develops Faster in Birds Than in Mammals,” General and Comparative Endocrinology 172, no. 1 (May 2011): 23–30. 21199656 10.1016/j.ygcen.2010.12.022

[20] A. Haim and A. E. Zubidat , “Artificial Light at Night: Melatonin as a Mediator Between the Environment and Epigenome,” Philosophical Transactions of the Royal Society, B: Biological Sciences 370, no. 1667 (May 2015): 20140121. 10.1098/rstb.2014.0121 PMC4375362 25780234

[21] J. S. Takahashi , H. Hamm , and M. Menaker , “Circadian Rhythms of Melatonin Release From Individual Superfused Chicken Pineal Glands In Vitro,” Proceedings of the National Academy of Sciences 77, no. 4 (April 1980): 2319–2322. 10.1073/pnas.77.4.2319 PMC348706 6929552

[22] Y. Li and V. M. Cassone , “Clock‐Controlled Regulation of the Acute Effects of Norepinephrine on Chick Pineal Melatonin Rhythms,” Journal of Biological Rhythms 30, no. 6 (December 2015): 519–532. 26446873 10.1177/0748730415607060

[23] D. Nätt , N. Lindqvist , H. Stranneheim , J. Lundeberg , P. A. Torjesen , and P. Jensen , “Inheritance of Acquired Behaviour Adaptations and Brain Gene Expression in Chickens,” PLoS One 4, no. 7 (July 2009): e6405. 19636381 10.1371/journal.pone.0006405 PMC2713434

[24] C. Lindqvist and P. Jensen , “Domestication and Stress Effects on Contrafreeloading and Spatial Learning Performance in Red Jungle Fowl (*Gallus gallus*) and White Leghorn Layers,” Behavioural Processes 81, no. 1 (May 2009): 80–84. 19429200 10.1016/j.beproc.2009.02.005

[25] C. Lindqvist , A. M. Janczak , D. Nätt , et al., “Transmission of Stress‐Induced Learning Impairment and Associated Brain Gene Expression From Parents to Offspring in Chickens,” PLoS One 2, no. 4 (April 2007): e364. 17426812 10.1371/journal.pone.0000364 PMC1838921

[26] S. Doyle and M. Menaker , “Circadian Photoreception in Vertebrates,” Cold Spring Harbor Symposia on Quantitative Biology 72, no. 1 (January 2007): 499–508. 18419310 10.1101/sqb.2007.72.003

[27] J. Denham , F. Z. Marques , E. L. Bruns , B. J. O'Brien , and F. J. Charchar , “Epigenetic Changes in Leukocytes After 8 Weeks of Resistance Exercise Training,” European Journal of Applied Physiology 116, no. 6 (2016): 1245–1253, http://www.ncbi.nlm.nih.gov/pubmed/27155847. 27155847 10.1007/s00421-016-3382-2

[28] R. Feil and M. F. Fraga , “Epigenetics and the Environment: Emerging Patterns and Implications,” Nature Reviews Genetics 13, no. 2 (January 2012): 97–109. 10.1038/nrg3142 22215131

[29] A. Bird , “DNA Methylation Patterns and Epigenetic Memory,” Genes & Development 16, no. 1 (January 2002): 6–21. 11782440 10.1101/gad.947102

[30] M. K. Skinner , M. Manikkam , and C. Guerrero‐Bosagna , “Epigenetic Transgenerational Actions of Environmental Factors in Disease Etiology,” Trends in Endocrinology and Metabolism: TEM 21, no. 4 (April 2010): 214–222. 20074974 10.1016/j.tem.2009.12.007 PMC2848884

[31] S. R. Friedrich , A. A. Nevue , A. L. P. Andrade , T. A. F. Velho , and C. V. Mello , “Emergence of Sex‐Specific Transcriptomes in a Sexually Dimorphic Brain Nucleus,” Cell Reports 40, no. 5 (August 2022): 111152. 35926465 10.1016/j.celrep.2022.111152 PMC9385264

[32] D. Holoch and D. Moazed , “RNA‐Mediated Epigenetic Regulation of Gene Expression,” Nature Reviews Genetics 16, no. 2 (February 2015): 71–84. 10.1038/nrg3863 PMC4376354 25554358

[33] C. A. Cornil and J. Balthazart , “Contribution of Birds to the Study of Sexual Differentiation of Brain and Behavior,” Hormones and Behavior 155 (September 2023): 105410. 37567061 10.1016/j.yhbeh.2023.105410 PMC10543621

[34] S. Naurin , B. Hansson , D. Hasselquist , Y. H. Kim , and S. Bensch , “The Sex‐Biased Brain: Sexual Dimorphism in Gene Expression in Two Species of Songbirds,” BMC Genomics 12, no. 1 (December 2011): 37. 21235773 10.1186/1471-2164-12-37 PMC3036617

[35] J. O. Valdebenito , K. H. Maher , G. Zachár , et al., “Sex Differences in Immune Gene Expression in the Brain of a Small Shorebird,” Immunogenetics 74, no. 5 (October 2022): 487–496. 35084547 10.1007/s00251-022-01253-w PMC8792134

[36] J. Bélteky , B. Agnvall , L. Bektic , A. Höglund , P. Jensen , and C. Guerrero‐Bosagna , “Epigenetics and Early Domestication: Differences in Hypothalamic DNA Methylation Between Red Junglefowl Divergently Selected for High or Low Fear of Humans,” Genetics, Selection, Evolution 50, no. 1 (December 2018): 13, https://gsejournal.biomedcentral.com/articles/10.1186/s12711-018-0384-z. 10.1186/s12711-018-0384-z PMC5880090 29609558

[37] J. C. Dunlap , “Molecular Bases for Circadian Clocks,” Cell 96, no. 2 (January 1999): 271–290. 9988221 10.1016/s0092-8674(00)80566-8

[38] X. Ding , T. Pan , Q. Tian , et al., “Profiling Temporal Changes of the Pineal Transcriptomes at Single Cell Level Upon Neonatal HIBD,” Frontiers in Cell and Developmental Biology 10 (March 2022): 794012, 10.3389/fcell.2022.794012. 35350377 PMC8958010

[39] J. C. Mays , M. C. Kelly , S. L. Coon , et al., “Single‐Cell RNA Sequencing of the Mammalian Pineal Gland Identifies Two Pinealocyte Subtypes and Cell Type‐Specific Daily Patterns of Gene Expression,” PLoS One 13, no. 10 (October 2018): e0205883. 30347410 10.1371/journal.pone.0205883 PMC6197868

[40] C. Li , X. He , Z. Zhang , C. Ren , and M. Chu , “Pineal Gland Transcriptomic Profiling Reveals the Differential Regulation of lncRNA and mRNA Related to Prolificacy in STH Sheep With Two FecB Genotypes,” BMC Genomic Data 22, no. 1 (December 2021): 9. 33602139 10.1186/s12863-020-00957-w PMC7893892

[41] Y. Yang , R. Zhou , W. Li , et al., “Dynamic Transcriptome Analysis Reveals Potential Long Non‐Coding RNAs Governing Postnatal Pineal Development in Pig,” Frontiers in Genetics 10 (May 2019): 409, 10.3389/fgene.2019.00409. 31130986 PMC6510172

[42] A. Korkmaz and R. J. Reiter , “Epigenetic Regulation: A New Research Area for Melatonin?,” Journal of Pineal Research 44, no. 1 (January 2008): 41–44. 18078446 10.1111/j.1600-079X.2007.00509.x

[43] H. Ellegren and J. Parsch , “The Evolution of Sex‐Biased Genes and Sex‐Biased Gene Expression,” Nature Reviews Genetics 8, no. 9 (September 2007): 689–698. 10.1038/nrg2167 17680007

[44] B. C. Bobotis , O. Braniff , M. Gargus , E. T. Akinluyi , I. O. Awogbindin , and M. È. Tremblay , “Sex Differences of Microglia in the Healthy Brain From Embryonic Development to Adulthood and Across Lifestyle Influences,” Brain Research Bulletin 202 (October 2023): 110752. 37652267 10.1016/j.brainresbull.2023.110752

[45] B. C. Bobotis , M. Khakpour , O. Braniff , et al., “Sex Chromosomes and Sex Hormones Differently Shape Microglial Properties During Normal Physiological Conditions in the Adult Mouse Hippocampus,” Journal of Neuroinflammation 22, no. 1 (January 2025): 18. 39856696 10.1186/s12974-025-03341-6 PMC11762133

[46] J. Lees , F. Pèrtille , P. Løtvedt , P. Jensen , and C. G. Bosagna , “The Mitoepigenome Responds to Stress, Suggesting Novel Mito‐Nuclear Interactions in Vertebrates,” BMC Genomics 24, no. 1 (September 2023): 561, https://bmcgenomics.biomedcentral.com/articles/10.1186/s12864-023-09668-9. 37736707 10.1186/s12864-023-09668-9 PMC10515078

[47] A. M. Bolger , M. Lohse , and B. Usadel , “Trimmomatic: A Flexible Trimmer for Illumina Sequence Data,” Bioinformatics 30, no. 15 (August 2014): 2114–2120. 24695404 10.1093/bioinformatics/btu170 PMC4103590

[48] A. Dobin , C. A. Davis , F. Schlesinger , et al., “STAR: Ultrafast Universal RNA‐Seq Aligner,” Bioinformatics 29, no. 1 (January 2013): 15–21. 23104886 10.1093/bioinformatics/bts635 PMC3530905

[49] M. Pertea , G. M. Pertea , C. M. Antonescu , T. C. Chang , J. T. Mendell , and S. L. Salzberg , “StringTie Enables Improved Reconstruction of a Transcriptome From RNA‐Seq Reads,” Nature Biotechnology 33 (February 2015): 290–295. 10.1038/nbt.3122 PMC4643835 25690850

[50] Y. Liao , G. K. Smyth , and W. Shi , “featureCounts: An Efficient General Purpose Program for Assigning Sequence Reads to Genomic Features,” Bioinformatics 30, no. 7 (April 2014): 923–930. 24227677 10.1093/bioinformatics/btt656

[51] B. Langmead and S. L. Salzberg , “Fast Gapped‐Read Alignment With Bowtie 2,” Nature Methods 9, no. 4 (March 2012): 357–359. 22388286 10.1038/nmeth.1923 PMC3322381

[52] H. Li , B. Handsaker , A. Wysoker , et al., “The Sequence Alignment/Map Format and SAMtools,” Bioinformatics 25, no. 16 (August 2009): 2078–2079. 19505943 10.1093/bioinformatics/btp352 PMC2723002

[53] F. Pértille , M. Brantsæter , J. Nordgreen , et al, “DNA Methylation Profiles in Red Blood Cells of Adult Hens Correlate to Their Rearing Conditions,” Journal of Experimental Biology [Internet] 220, no. 19 (January 2017): 3579–3587, http://jeb.biologists.org/lookup/doi/10.1242/jeb.157891. 28784681 10.1242/jeb.157891

[54] M. D. Robinson and A. Oshlack , “A Scaling Normalization Method for Differential Expression Analysis of RNA‐Seq Data,” Genome Biology 11, no. 3 (2010): R25. 20196867 10.1186/gb-2010-11-3-r25 PMC2864565

[55] R Development Core Team R , “R: A Language and Environment for Statistical Computing,” in R Foundation for Statistical Computing, ed. Team RDC (R Foundation for Statistical Computing, 2011), 409.

[56] G. Yu , L. G. Wang , and Q. Y. He , “ChIPseeker: An R/Bioconductor Package for ChIP Peak Annotation, Comparison and Visualization,” Bioinformatics 31, no. 14 (July 2015): 2382–2383, https://academic.oup.com/bioinformatics/article-lookup/doi/10.1093/bioinformatics/btv145. 25765347 10.1093/bioinformatics/btv145

[57] S. D. Ghiassian , J. Menche , and A. L. Barabási , “A DIseAse MOdule Detection (DIAMOnD) Algorithm Derived From a Systematic Analysis of Connectivity Patterns of Disease Proteins in the Human Interactome,” PLoS Computational Biology 11, no. 4 (April 2015): e1004120. 25853560 10.1371/journal.pcbi.1004120 PMC4390154

[58] C. von Mering , L. J. Jensen , M. Kuhn , et al., “STRING 7—Recent Developments in the Integration and Prediction of Protein Interactions,” Nucleic Acids Research 35 (January 2007): D358–D362. 17098935 10.1093/nar/gkl825 PMC1669762

[59] D. Szklarczyk , A. L. Gable , D. Lyon , et al., “STRING v11: Protein–Protein Association Networks With Increased Coverage, Supporting Functional Discovery in Genome‐Wide Experimental Datasets,” Nucleic Acids Research 47, no. D1 (January 2019): D607–D613. 30476243 10.1093/nar/gky1131 PMC6323986

[60] M. E. Smoot , K. Ono , J. Ruscheinski , P. L. Wang , and T. Ideker , “Cytoscape 2.8: New Features for Data Integration and Network Visualization,” Bioinformatics 27, no. 3 (February 2011): 431–432. 21149340 10.1093/bioinformatics/btq675 PMC3031041

[61] J. Reimand , T. Arak , P. Adler , et al., “G:Profiler—A Web Server for Functional Interpretation of Gene Lists (2016 Update),” Nucleic Acids Research 44, no. W1 (July 2016): W83–W89. 27098042 10.1093/nar/gkw199 PMC4987867

[62] H. A. de Weerd, , T. V. S. Badam , D. Martínez‐Enguita , et al., “MODifieR: An Ensemble R Package for Inference of Disease Modules From Transcriptomics Networks,” Bioinformatics 36 (April 2020): 3918–3919. 32271876 10.1093/bioinformatics/btaa235

[63] N. Goel and T. L. Bale , “Examining the Intersection of Sex and Stress in Modelling Neuropsychiatric Disorders,” Journal of Neuroendocrinology 21, no. 4 (April 2009): 415–420. 19187468 10.1111/j.1365-2826.2009.01843.x PMC2716060

[64] T. L. Bale , “Sex Differences in Prenatal Epigenetic Programing of Stress Pathways,” Stress (Amsterdam) 14, no. 4 (July 2011): 348–356. 10.3109/10253890.2011.586447 21663536

[65] D. J. Cosgrove , Z. Gordon , J. E. Bernie , et al., “Sexual Dysfunction in Combat Veterans With Post‐Traumatic Stress Disorder,” Urology (Ridgewood NJ) 60, no. 5 (November 2002): 881–884. 10.1016/s0090-4295(02)01899-x 12429320

[66] M. Hines , K. J. Johnston , S. Golombok , J. Rust , M. Stevens , and J. Golding , “Prenatal Stress and Gender Role Behavior in Girls and Boys: A Longitudinal, Population Study,” Hormones and Behavior 42, no. 2 (September 2002): 126–134. 12367566 10.1006/hbeh.2002.1814

[67] T. Allen and B. Haggett , “Group Housing of Pregnant Mice Reduces Copulatory Receptivity of Female Progeny,” Physiology & Behavior 19, no. 1 (July 1977): 61–68. 11803692 10.1016/0031-9384(77)90160-3

[68] N. Sachser and S. Kaiser , “Prenatal Social Stress Masculinizes the Females' Behaviour in Guinea Pigs,” Physiology & Behavior 60, no. 2 (August 1996): 589–594. 8840923 10.1016/s0031-9384(96)80036-9

[69] N. G. Forger , “Past, Present and Future of Epigenetics in Brain Sexual Differentiation,” Journal of Neuroendocrinology 30, no. 2 (February 2018): e12492. 10.1111/jne.12492 28585265

[70] H. Ellegren , “Dosage Compensation: Do Birds Do It as Well?,” Trends in Genetics 18, no. 1 (January 2002): 25–28. 11750697 10.1016/s0168-9525(01)02553-7

[71] A. M. Livernois , S. A. Waters , J. E. Deakin , J. A. Marshall Graves , and P. D. Waters , “Independent Evolution of Transcriptional Inactivation on Sex Chromosomes in Birds and Mammals,” PLoS Genetics 9, no. 7 (July 2013): e1003635. 23874231 10.1371/journal.pgen.1003635 PMC3715422

[72] S. M. Gartler and A. D. Riggs , “Mammalian X‐Chromosome Inactivation,” Annual Review of Genetics 17, no. 1 (December 1983): 155–190. 10.1146/annurev.ge.17.120183.001103 6364959

[73] M. Crone , J. C. Jensenius , and C. Koch , “B‐L Antigens (Ia‐Like Antigens) of the Chicken Major Histocompatibility Complex,” Scandinavian Journal of Immunology 14, no. 6 (December 1981): 591–597. 6210953 10.1111/j.1365-3083.1981.tb00600.x

[74] E. van de Vosse and J. T. van Dissel , “IFN‐γR1 Defects: Mutation Update and Description of the IFNGR1 Variation Database,” Human Mutation 38, no. 10 (October 2017): 1286–1296. 28744922 10.1002/humu.23302

[75] X. Lin , J. Wang , L. Yun , et al., “Association Between LEKR1‐CCNL1 and IGSF21‐KLHDC7A Gene Polymorphisms and Diabetic Retinopathy of Type 2 Diabetes Mellitus in the Chinese Han Population,” Journal of Gene Medicine 18, no. 10 (October 2016): 282–287. 27607899 10.1002/jgm.2926

[76] A. M. Deaton and A. Bird , “CpG Islands and the Regulation of Transcription,” Genes & Development 25, no. 10 (May 2011): 1010–1022. 21576262 10.1101/gad.2037511 PMC3093116

[77] P. A. Jones , “The DNA Methylation Paradox,” Trends in Genetics 15, no. 1 (January 1999): 34–37, http://www.ncbi.nlm.nih.gov/pubmed/10087932. 10087932 10.1016/s0168-9525(98)01636-9

[78] A. Meissner , T. S. Mikkelsen , H. Gu , et al., “Genome‐Scale DNA Methylation Maps of Pluripotent and Differentiated Cells,” Nature 454, no. 7205 (August 2008): 766–770. 18600261 10.1038/nature07107 PMC2896277

[79] G. Lev Maor , A. Yearim , and G. Ast , “The Alternative Role of DNA Methylation in Splicing Regulation,” Trends in Genetics 31, no. 5 (May 2015): 274–280. 25837375 10.1016/j.tig.2015.03.002

[80] M. B. Stadler , R. Murr , L. Burger , et al., “DNA‐Binding Factors Shape the Mouse Methylome at Distal Regulatory Regions,” Nature 480, no. 7378 (December 2011): 490–495. 22170606 10.1038/nature10716

[81] C. Luo , P. Hajkova , and J. R. Ecker , “Dynamic DNA Methylation: In the Right Place at the Right Time,” Science (1979) 361, no. 6409 (September 2018): 1336–1340. 10.1126/science.aat6806 PMC6197482 30262495

[82] Y. Song , P. R. van den Berg , S. Markoulaki , et al., “Dynamic Enhancer DNA Methylation as Basis for Transcriptional and Cellular Heterogeneity of ESCs,” Molecular Cell 75, no. 5 (September 2019): 905–920.e6. 31422875 10.1016/j.molcel.2019.06.045 PMC6731151

[83] L. A. Pennacchio , W. Bickmore , A. Dean , M. A. Nobrega , and G. Bejerano , “Enhancers: Five Essential Questions,” Nature Reviews Genetics 14, no. 4 (April 2013): 288–295. 10.1038/nrg3458 PMC4445073 23503198

[84] G. A. Maston , S. K. Evans , and M. R. Green , “Transcriptional Regulatory Elements in the Human Genome,” Annual Review of Genomics and Human Genetics 7, no. 1 (September 2006): 29–59. 10.1146/annurev.genom.7.080505.115623 16719718

[85] H. Tipney and L. Hunter , “An Introduction to Effective Use of Enrichment Analysis Software,” Human Genomics 4, no. 3 (February 2010): 202–206. 20368141 10.1186/1479-7364-4-3-202 PMC3525973

[86] B. Xiao , T. M. Chen , and Y. Zhong , “Possible Molecular Mechanism Underlying Cadmium‐Induced Circadian Rhythms Disruption in Zebrafish,” Biochemical and Biophysical Research Communications 481, no. 3–4 (December 2016): 201–205. 27784643 10.1016/j.bbrc.2016.10.081

[87] Y. Wang , K. Lv , M. Zhao , et al., “Expression Profiles and Functional Annotation Analysis of mRNAs in Suprachiasmatic Nucleus of Clock Mutant Mice,” Gene 647 (March 2018): 107–114. 29307853 10.1016/j.gene.2017.12.056

[88] H. J. Bailes , N. Milosavljevic , L. Y. Zhuang , et al., “Optogenetic Interrogation Reveals Separable G‐Protein‐Dependent and ‐Independent Signalling Linking G‐Protein‐Coupled Receptors to the Circadian Oscillator,” BMC Biology 15, no. 1 (December 2017): 40. 28506231 10.1186/s12915-017-0380-8 PMC5430609

[89] J. S. O'Neill and A. B. Reddy , “The Essential Role of cAMP/Ca2+ Signalling in Mammalian Circadian Timekeeping,” Biochemical Society Transactions 40, no. 1 (February 2012): 44–50. 22260664 10.1042/BST20110691 PMC3399769

[90] C. Fukuhara , C. Liu , T. N. Ivanova , et al., “Gating of the cAMP Signaling Cascade and Melatonin Synthesis by the Circadian Clock in Mammalian Retina,” Journal of Neuroscience 24, no. 8 (February 2004): 1803–1811. 14985420 10.1523/JNEUROSCI.4988-03.2004 PMC6730387

[91] W. W. Hwang‐Verslues , P. H. Chang , Y. M. Jeng , et al., “Loss of Corepressor PER2 Under Hypoxia Up‐Regulates OCT1‐Mediated EMT Gene Expression and Enhances Tumor Malignancy,” Proceedings of the National Academy of Sciences 110, no. 30 (July 2013): 12331–12336. 10.1073/pnas.1222684110 PMC3725072 23836662

[92] R. Balasubramanian , D. A. Cohen , E. B. Klerman , et al., “Absence of Central Circadian Pacemaker Abnormalities in Humans With Loss of Function Mutation in Prokineticin 2,” Journal of Clinical Endocrinology & Metabolism 99, no. 3 (March 2014): E561–E566. 24423319 10.1210/jc.2013-2096 PMC3942237

[93] Q. Y. Zhou and M. Y. Cheng , “Prokineticin 2 and Circadian Clock Output,” FEBS Journal 272, no. 22 (November 2005): 5703–5709. 16279936 10.1111/j.1742-4658.2005.04984.x PMC2667323

[94] S. Moravcová , D. Pačesová , B. Melkes , et al., “The Day/Night Difference in the Circadian Clock's Response to Acute Lipopolysaccharide and the Rhythmic Stat3 Expression in the Rat Suprachiasmatic Nucleus,” PLoS One 13, no. 9 (September 2018): e0199405. 30265676 10.1371/journal.pone.0199405 PMC6161871

[95] J. A. Ripperger , S. Fritz , K. Richter , G. M. Hocke , F. Lottspeich , and G. H. Fey , “Transcription Factors Stat3 and Stat5b Are Present in Rat Liver Nuclei Late in an Acute Phase Response and Bind Interleukin‐6 Response Elements,” Journal of Biological Chemistry 270, no. 50 (December 1995): 29998–30006. 8530402 10.1074/jbc.270.50.29998

[96] L. Hein , J. D. Altman , and B. K. Kobilka , “Two Functionally Distinct α2‐Adrenergic Receptors Regulate Sympathetic Neurotransmission,” Nature 402, no. 6758 (November 1999): 181–184. 10647009 10.1038/46040

[97] Y. Mao and S. C. Finnemann , “Essential Diurnal Rac1 Activation During Retinal Phagocytosis Requires αvβ5 Integrin but Not Tyrosine Kinases Focal Adhesion Kinase or Mer Tyrosine Kinase,” Molecular Biology of the Cell 23, no. 6 (March 2012): 1104–1114. 22262456 10.1091/mbc.E11-10-0840 PMC3302737

[98] A. A. H. Ali , G. A. Avakian , and C. Von Gall , “The Role of Purinergic Receptors in the Circadian System,” International Journal of Molecular Sciences 21, no. 10 (May 2020): 3423. 32408622 10.3390/ijms21103423 PMC7279285

[99] M. Zeidler and C. G. Kleer , “The Polycomb Group Protein Enhancer of Zeste 2: Its Links to DNA Repair and Breast Cancer,” Journal of Molecular Histology 37, no. 5–7 (November 2006): 219–223. 16855786 10.1007/s10735-006-9042-9

[100] C. Y. Chen , R. W. Logan , T. Ma , et al., “Effects of Aging on Circadian Patterns of Gene Expression in the Human Prefrontal Cortex,” Proceedings of the National Academy of Sciences 113, no. 1 (January 2016): 206–211. 10.1073/pnas.1508249112 PMC4711850 26699485

[101] N. Matosin , K. A. Newell , Y. Quidé , et al., “Effects of Common GRM5 Genetic Variants on Cognition, Hippocampal Volume and mGluR5 Protein Levels in Schizophrenia,” Brain Imaging and Behavior 12, no. 2 (April 2018): 509–517. 28405888 10.1007/s11682-017-9712-0

[102] L. M. Guissoni Campos , A. Hataka , I. Z. Vieira , et al., “Circadian Clock Proteins and Melatonin Receptors in Neurons and Glia of the *Sapajus apella* Cerebellum,” Frontiers in Physiology 9 (February 2018): 5, 10.3389/fphys.2018.00005. 29479318 PMC5811497

[103] G. R. Yamakawa , P. Basu , F. Cortese , et al., “The Cholinergic Forebrain Arousal System Acts Directly on the Circadian Pacemaker,” Proceedings of the National Academy of Sciences 113, no. 47 (November 2016): 13498–13503. 10.1073/pnas.1610342113 PMC5127341 27821764

[104] R. A. Hut and E. A. Van der Zee , “The Cholinergic System, Circadian Rhythmicity, and Time Memory,” Behavioural Brain Research 221, no. 2 (August 2011): 466–480. 21115064 10.1016/j.bbr.2010.11.039

[105] A. Woller and D. Gonze , “The Bird Circadian Clock: Insights From a Computational Model,” Journal of Biological Rhythms 28, no. 6 (December 2013): 390–402. 24336417 10.1177/0748730413512454

[106] C. Fekete and R. M. Lechan , “Central Regulation of Hypothalamic‐Pituitary‐Thyroid Axis Under Physiological and Pathophysiological Conditions,” Endocrine Reviews 35, no. 2 (April 2014): 159–194. 24423980 10.1210/er.2013-1087 PMC3963261

