# Supplementary figures and images for: Sex‐Specific Methylomic and Transcriptomic Responses of the Avian Pineal Gland to Unpredictable Illumination Patterns
Source: J Pineal Res. 2025 Mar 17;77(2):e70040. doi: 10.1111/jpi.70040 (PMC11911909; doi:10.1111/jpi.70040)

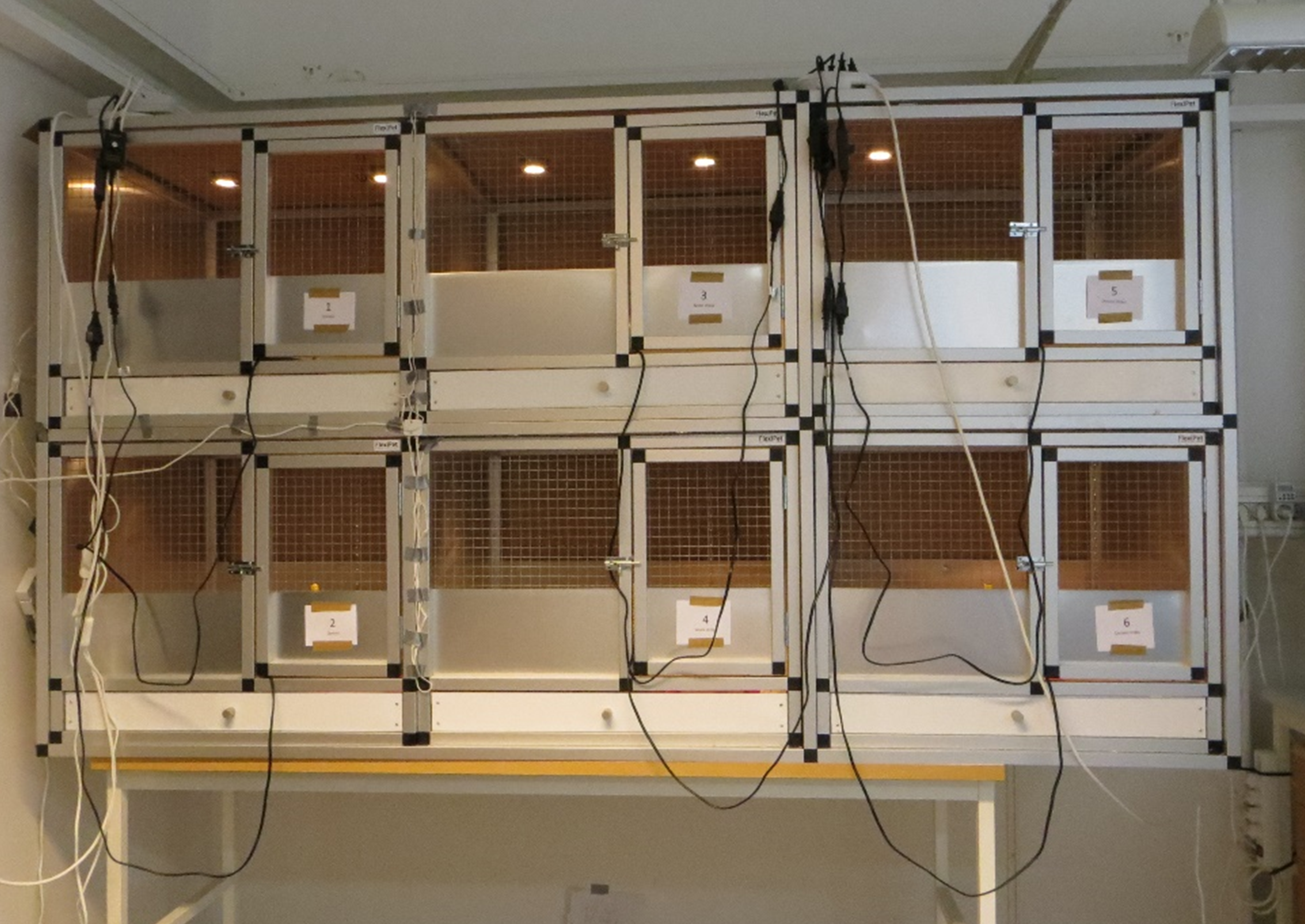

Supplement: Supplementary file 8 — Supporting information. [file JPI-77-e70040-s005.tif]

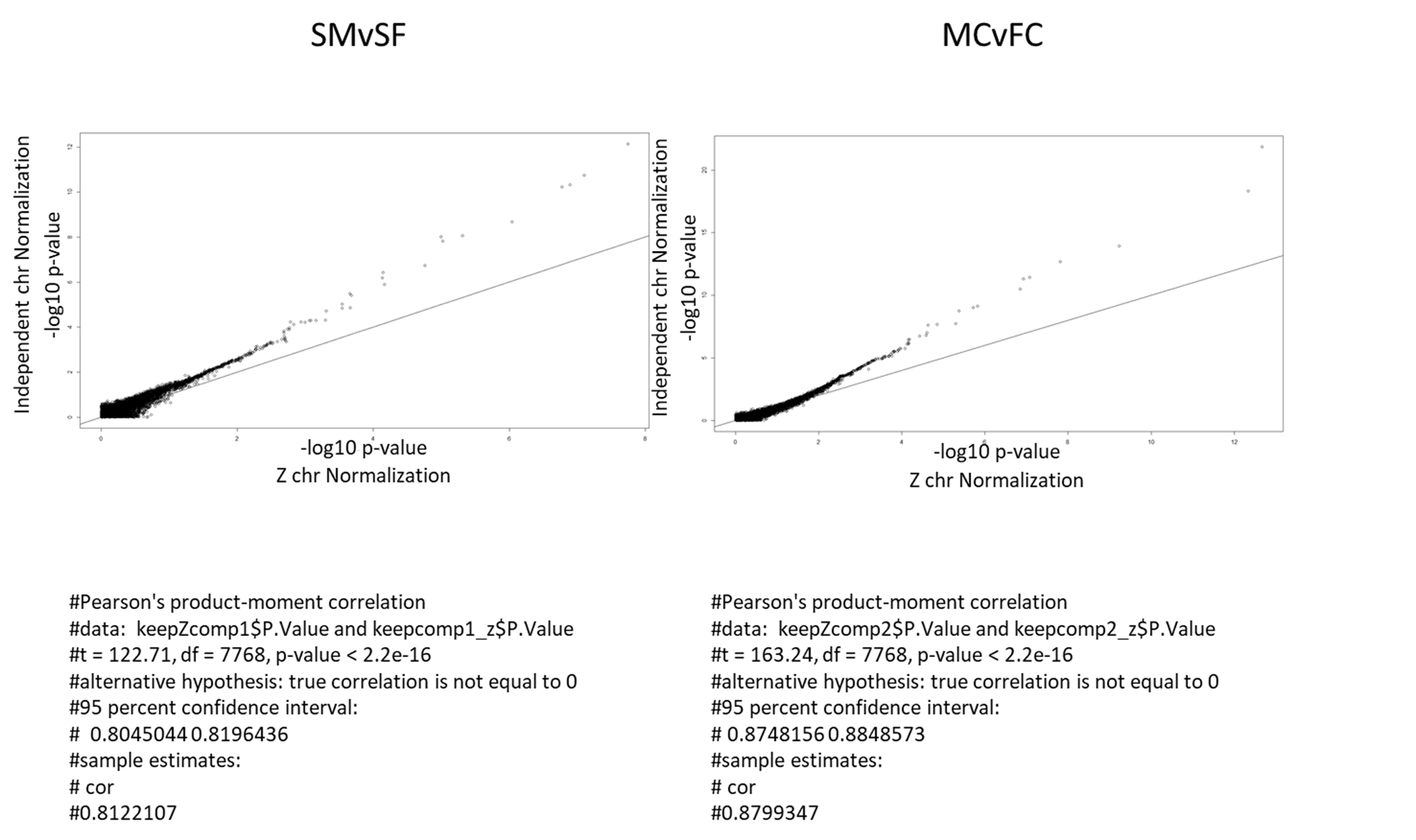

Supplement: Supplementary file 9 — Supporting information. [file JPI-77-e70040-s008.tif]

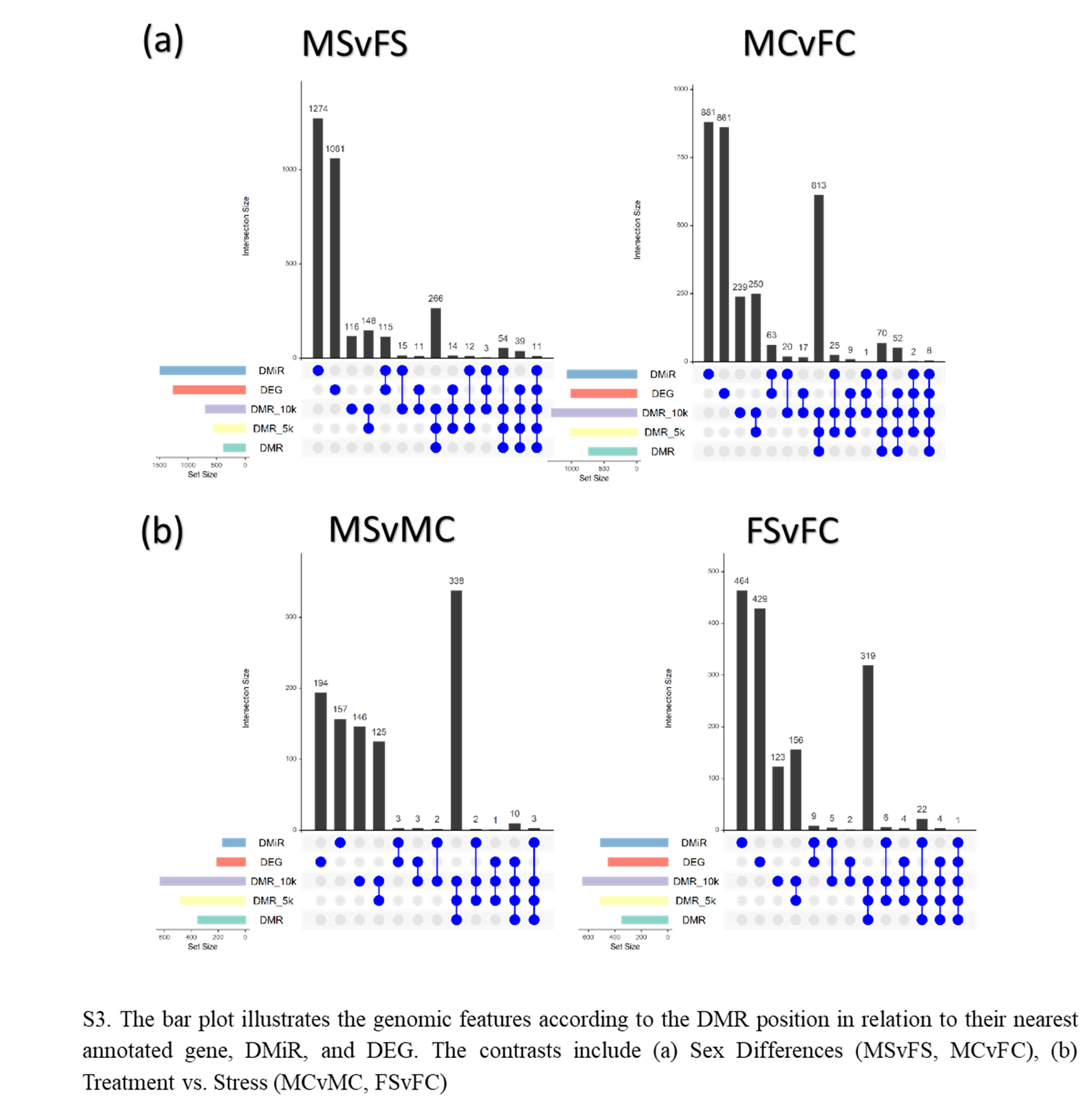

Supplement: Supplementary file 10 — Supporting information. [file JPI-77-e70040-s006.tif]
