# Supplementary material for: Sex‐Specific Methylomic and Transcriptomic Responses of the Avian Pineal Gland to Unpredictable Illumination Patterns
Source: J Pineal Res. 2025 Mar 17;77(2):e70040. doi: 10.1111/jpi.70040 (PMC11911909; doi:10.1111/jpi.70040)
Supplement: Supplementary file 11 — Supporting information. [file JPI-77-e70040-s003.docx]

**Sex-specific Methylomic and Transcriptomic Responses of the Avian Pineal Gland to Unpredictable Illumination Patterns**

**Running title:** Sex-specific Epigenomic Regulation in Bird Pineal Gland under Light Stress

Fábio Pértille^1, 2, 3^*****, Tejaswi Badam^4,5^, Nina Mitheiss^2^, Pia Løtvedt^2^, Emmanouil Tsakoumis^3^, Mika Gustafsson^4^, Luiz Lehmann Coutinho^1^, Per Jensen^2^ and Carlos Guerrero-Bosagna^2,3^*****

**SUPLEMENTARY TABLES:**

Supplementary Table S1. Comprehensive overview of omic features analyzed: differentially expressed genes (DEG), differentially methylated regions (DMR), and targets of differentially expressed microRNAs (DMiR), categorized by two thresholds of significance.

| **Omic** | **Total Features** | **MSvFS** | | **MCvFC** | | **MSvMC** | | **FSvFC** | | **SvC** | |
| --- | --- | --- | --- | --- | --- | --- | --- | --- | --- | --- | --- |
|  |  | Padj≤0.2 | P≤  0.05 | Padj≤0.2 | P≤  0.05 | Padj≤0.2 | P≤  0.05 | Padj≤0.2 | P≤  0.05 | Padj≤0.2 | P≤  0.05 |
| **DEG** | 14492 genes | 111 | 1254 | 64 | 1011 | 0 | 211 | 0 | 449 | 0 | 480 |
| **DMR** | 37382 regions | 47 | 2217 | 1284 | 4245 | 6 | 1542 | 17 | 1891 | 2 | 1076 |
|  | Within Genes | 3 | 371 | 229 | 742 | 1 | 352 | 4 | 347 | 0 | 303 |
|  | Gene TSS- 5K | 1 | 174 | 89 | 286 | 0 | 128 | 2 | 166 | 0 | 134 |
|  | Gene 5K-10K | 1 | 145 | 88 | 277 | 0 | 151 | 2 | 130 | 0 | 95 |
| **DMiR** | 352(miRNA) | 0 | 26 | 0 | 17 | 0 | 8 | 0 | 11 | 0 | 10 |
|  | Target genes (conf>90%) | 0 | 1484 | 0 | 1068 | 0 | 167 | 0 | 507 | 0 | 468 |
|  | Target genes (conf>99%) | 0 | 176 | 0 | 67 | 0 | 1 | 0 | 31 | 0 | 25 |

* MSvFS and MCvFC represent the sex comparison within the Stress and Control groups, respectively; MCvMC and FSvFC compare Stress versus Control within females and males, respectively; SvC evaluates the effect of stress versus control, incorporating sex as a fixed variable in the model (sex-fixed model). The adjusted P-value for multiple comparisons was corrected using FDR analysis. DMR-associated genes were identified within genes, between gene transcription start and stop (TSS) sites and within 5 Kb, and between 5 and 10Kb from the TSS. Two prediction score confidence thresholds are presented for the analysis of miRNA target genes.
