# Supplementary material for: Sex‐Specific Methylomic and Transcriptomic Responses of the Avian Pineal Gland to Unpredictable Illumination Patterns
Source: J Pineal Res. 2025 Mar 17;77(2):e70040. doi: 10.1111/jpi.70040 (PMC11911909; doi:10.1111/jpi.70040)
Supplement: Supplementary file 1 — Supporting information. [file JPI-77-e70040-s007.docx]

**Supplementary Materials for the Manuscript**
*Sex-specific Methylomic and Transcriptomic Responses of the Avian Pineal Gland to Unpredictable Illumination Patterns*

Dear Editor,

Due to the large size of the supplementary materials and scripts associated with this manuscript, we are providing a direct link to GitHub where all relevant data is hosted. This includes the supplementary spreadsheets, figures, and detailed analysis scripts that ensure reproducibility of the study.

You can access all the supplementary materials and scripts here:
<https://github.com/fpertille/PinealGlandChicken>

The repository contains:

1. Supplementary Spreadsheets (S1-S6) and Figures (S1, S2, S3)
2. Scripts for statistical analysis, input generation, and graph/table creation
3. Details for replicating the study's omic-level analysis (DMR, DMiR, DEG) using the Limma package

Should there be any issues accessing the files or if further details are needed, please feel free to reach out.

Thank you for your understanding.

Best regards,
Fábio Pértille
Uppsala University
fabio.pertille@ebc.uu.se
